# MRN–CtIP, EXO1, and DNA2–WRN/BLM act bidirectionally to process DNA gaps in PARPi-treated cells without strand cleavage

**DOI:** 10.1101/gad.352421.124

**Published:** 2025-05-01

**Authors:** Isabelle M. Seppa, Ilaria Ceppi, Mithila Tennakoon, Giordano Reginato, Jessica Jackson, Celia D. Rouault, Sumedha Agashe, Vladislav O. Sviderskiy, Mangsi Limbu, Erica Lantelme, Alice Meroni, Stefan Braunshier, Damiano Borrello, Priyanka Verma, Petr Cejka, Alessandro Vindigni

**Affiliations:** 1 Division of Oncology, Department of Medicine, Washington University in St. Louis, St. Louis, Missouri 63110, USA;; 2 Institute for Research in Biomedicine (IRB), Università della Svizzera italiana, CH 6500 Bellinzona, Switzerland;; 3 Department of Pathology and Immunology, Washington University School of Medicine, St. Louis, Missouri 63110, USA

**Keywords:** BRCA, DNA replication, DNA replication stress, MRE11, PARP inhibitor, genome stability, nucleases, single-stranded DNA gaps

## Abstract

Single-stranded DNA (ssDNA) gaps impact genome stability and PARP inhibitor (PARPi) sensitivity, especially in BRCA1/2-deficient tumors. Using single-molecule DNA fiber analysis, electron microscopy, and biochemical methods, we found that MRN, CtIP, EXO1, and DNA2–WRN/BLM resect ssDNA gaps through a mechanism different from their actions at DNA ends. MRN resects ssDNA gaps in the 3′-to-5′ direction using its pCtIP-stimulated exonuclease activity. Unlike at DNA ends, MRN does not use its endonucleolytic activity to cleave the 5′-terminated strand flanking the gap or the ssDNA. EXO1 and DNA2–WRN/BLM specifically resect the 5′ end of the gap independent of MRN–CtIP. This resection process alters ssDNA gap repair kinetics in BRCA1-proficient and -deficient cells. In BRCA1-deficient cells treated with PARPis, excessive resection results in larger ssDNA gaps, hindering their repair and leading to DNA breaks in subsequent cell cycle stages due to ssDNA gaps colliding with DNA replication forks. These findings broaden our understanding of the role of human nucleases in DNA metabolism and have significant implications for defining the mechanisms driving PARPi sensitivity in BRCA-deficient tumors.

## Abstract (precis)

In this study, Seppa et al. dissect the mechanisms by which DNA nucleases such as MRN–CtIP, EXO1, and DNA2–WRN/BLM resect single-strand DNA gaps and regulate repair kinetics in a manner distinct from their activity at double-strand DNA breaks. These mechanisms have critical implications for DNA repair and replication in BRCA1-deficient cells and for sensitivity to PARP inhibitors.

Single-stranded DNA (ssDNA) gaps are potentially genome-destabilizing structures that require prompt repair to prevent DNA breakage and genome instability. They can be located behind the replication fork on the daughter strands or at the replication fork junction itself. Fork junction gaps generally arise because of the uncoupling between the replicative polymerase and the replicative helicase upon replication fork stalling (Byun et al. 2005; Cortez 2019). Internal gaps behind the replication fork can form on the lagging strand because of defects in Okazaki fragment processing (Berti et al. 2020; Mann et al. 2022; Vaitsiankova et al. 2022). Conversely, internal leading strand gaps can form when DNA synthesis resumes downstream from replication-blocking lesions in a process termed repriming, which is mediated by the human primase and DNA-directed polymerase (PRIMPOL) in mammalian cells (Bianchi et al. 2013; García-Gómez et al. 2013; Mourón et al. 2013; Keen et al. 2014).

Recent research on the mechanisms needed to repair the internal gaps postreplicatively has uncovered two main DNA damage tolerance pathways in charge of this process: template switching (TS) and translesion synthesis (TLS) (Branzei and Szakal 2016; Piberger et al. 2020; Tirman et al. 2021). Furthermore, the Polθ polymerase, in addition to its well-established role in microhomology-mediated end joining (Brambati et al. 2020), was recently shown to be specifically required for repair of lagging strand ssDNA gaps (Belan et al. 2022; Mann et al. 2022; Schrempf et al. 2022). Our group, along with other laboratories, discovered that these ssDNA gap repair pathways are impaired in BRCA-deficient cells, explaining previous observations showing that ssDNA gaps accumulate in BRCA1-deficient tumors (Panzarino et al. 2021; Taglialatela et al. 2021; Tirman et al. 2021). Importantly, the aberrant accumulation of ssDNA gaps observed in the absence of BRCA proteins was recently linked to BRCA-deficient tumor sensitivity to therapies that target DNA, such as platinum-based compounds, or inhibit specific DNA repair pathways, such as PARP inhibitors (PARPis). These findings highlight the relevance of studying the mechanisms of ssDNA gap processing and repair in the context of cancer treatment (Cong et al. 2021; Paes Dias and Jonkers 2021; Paes Dias et al. 2021; Simoneau et al. 2021; Cong and Cantor 2022). Interestingly, inhibition of MRE11 activity can efficiently restore ssDNA gap repair in BRCA-deficient tumors, pointing to a central role for nucleases in the regulation of ssDNA gap processing and repair (Tirman et al. 2021).

The MRE11 nuclease possesses both a 3′–5′ exonuclease and endonuclease activity at 5′-terminated DNA strands near DNA breaks (Paull and Gellert 1998; Deshpande et al. 2016; Wang et al. 2017; Cannavo et al. 2019; Tamai et al. 2024). MRE11 is a component of the MRE11–RAD50–NBS1 (MRN) complex whose function has been largely studied at DNA ends in the context of double-strand break (DSB) repair (Lisby et al. 2004; Lamarche et al. 2010; Zhao et al. 2020). In the context of DSBs, DNA ends are resected in the 5′-to-3′ direction. Briefly, the MRN endonuclease activity, stimulated by CtIP, first nicks near the 5′-terminated ends at internal sites, which is followed by the 3′-to-5′ exonuclease activity of MRN that proceeds from the incision sites back toward the DNA end (Anand et al. 2016). In contrast, the long-range resection nucleases (EXO1 or DNA2) extend the resection tracts 5′ to 3′ in the opposite direction (Shibata et al. 2014; Cejka and Symington 2021). The same molecular details are not available for the function of MRE11 or MRN at ssDNA gaps. Recent studies suggested that the exonuclease and endonuclease activities of MRE11 are required for gap resection, with the endonuclease activity of MRE11 involved in cleaving the single-stranded segments of gaps, converting them into DSBs (Hale et al. 2023). However, the concept that gaps can be actively converted into a DSB by MRE11 or other nucleases remains controversial. Additional studies suggested that ssDNA gaps remain intact until the subsequent round of the cell cycle, where they are eventually converted into DSBs upon collision with an incoming replication fork (Simoneau et al. 2021). The long-range resection nucleases EXO1 and DNA2 were also implicated in ssDNA gap resection (Hale et al. 2023; García-Rodríguez et al. 2024; Nusawardhana et al. 2024), though their exact roles remain unclear.

Here, we combined single-molecule DNA fiber, electron microscopy (EM), and biochemical approaches to delineate how MRE11 (or MRN) and CtIP, alongside the long-range resection nucleases EXO1 and DNA2, process internal ssDNA gaps in PARPi-treated cells. Using EM, we precisely measured the size and location of the ssDNA gaps, as well as the effect of MRE11 inhibition on these parameters. We discovered that the mechanism by which MRE11, EXO1, and DNA2 nucleases process the ssDNA gaps is drastically different from the mechanism by which they process DSBs. MRN resects gaps using its 3′–5′ exonuclease activity that is stimulated by CtIP, whereas EXO1 and DNA2 nucleases resect gaps in the 5′–3′ direction. Unexpectedly, the endonuclease activity of MRN does not efficiently nick the DNA duplex adjacent to the gap or efficiently cleave the single-stranded region of the gap. Our findings suggest that the toxicity of ssDNA gaps in cells treated with PARPis is not associated with breaks directly resulting from nuclease-dependent ssDNA gap cleavage. Instead, it is related to breaks that form subsequently in the next cell cycle upon collision with DNA replication forks. These findings have significant implications for improving our understanding of the mechanisms driving PARPi sensitivity in BRCA-deficient cells and for identifying potential strategies to overcome PARPi resistance.

## Results

### Daughter strand ssDNA gaps are larger in BRCA1-deficient relative to BRCA1-proficient cells

**Figure 1.**
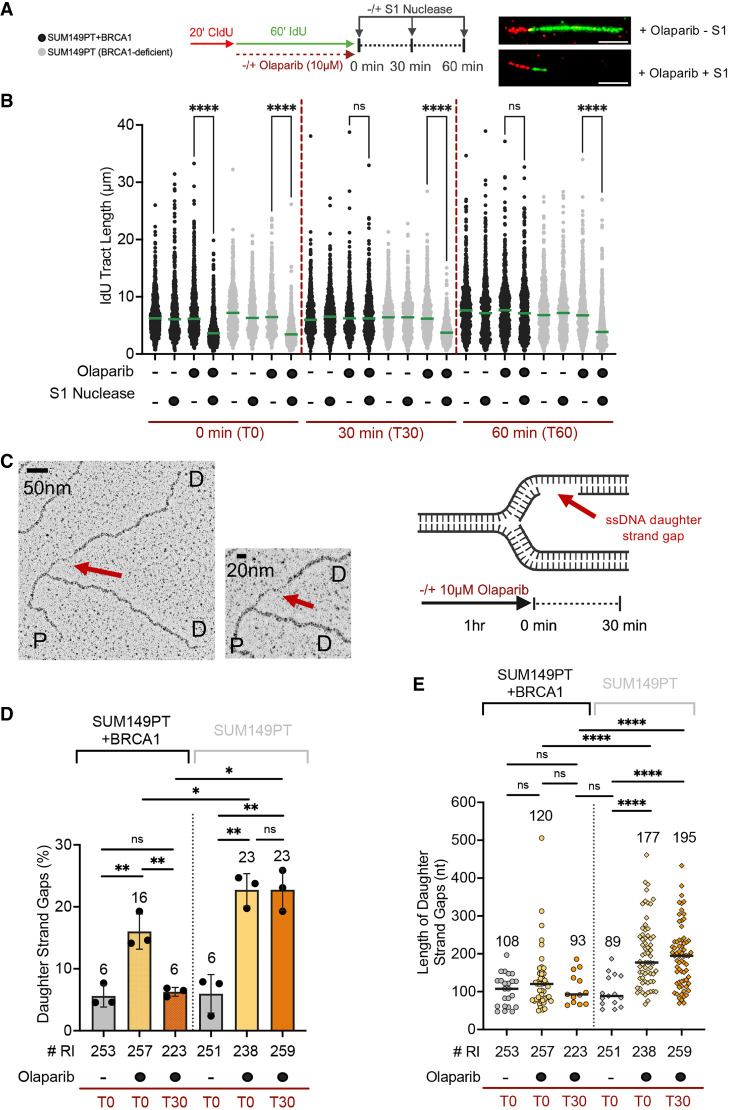
ssDNA gaps cannot be repaired in BRCA1-deficient cells treated with PARPis and are larger than in BRCA1-proficient cells (see also Supplemental Fig. S1). (*A*, *left*) Schematic of the DNA fiber spreading assay with the S1 nuclease. (*Right*) Representative images of DNA fibers in SUM149PT cells treated with 10 μM olaparib ± S1. Scale bar, 10 μm. (*B*) Dot plot and median of IdU tract lengths in SUM149PT and SUM149PT + BRCA1 cells ±10 μM olaparib (1 h) and ±S1. The S1 nuclease was added immediately after (0 min [T0]) and 30 min (T30) and 60 min (T60) after olaparib removal (*n* = 3). At least 180 tracts were scored for each sample. Statistics: Kruskal–Wallis followed by Dunn's multiple comparisons test. (ns) Nonsignificant, (****) *P* < 0.0001. (*C*, *left*) Representative electron micrograph of a replication fork containing an internal daughter strand ssDNA gap. (*Middle*) Magnified image of the fork junction. Red arrows indicate the daughter strand ssDNA gap. (P) Parental strand, (D) daughter strand. (*Right*) Schematic of the electron microscopy experiments of *D* and *E* and of a replication fork containing an internal gap. (*D*) Percentage of replication forks with daughter strand gaps in SUM149PT + BRCA1 and SUM149PT cells ±10 μM olaparib for 1 h. Cells were collected immediately after (T0) and 30 min (T30) after PARPi removal. “# RI” indicates the number of analyzed replication intermediates. (*n* = 3). Mean values are shown *above* each data set. Columns indicate mean ± SD. Statistics: unpaired *t*-test. (ns) Nonsignificant, (*) *P* < 0.0332, (**) *P* < 0.0021. (*E*) Length of daughter strand ssDNA gaps in nucleotides in SUM149PT and SUM149PT + BRCA1 cells treated as in *D*. “# RI” indicates the number of analyzed replication intermediates. (*n* = 3). Statistics: unpaired *t*-test with Welch correction. (ns) Nonsignificant, (****) *P* < 0.0001. Horizontal bars indicate median. Median values are shown *above* each data set.

Previous studies highlighted how ssDNA gaps accumulate in BRCA1-deficient tumors upon treatment with PARPis and various DNA lesion-inducing agents (Cong et al. 2021; Paes Dias et al. 2021; Panzarino et al. 2021; Simoneau et al. 2021). Here, we treated the *BRCA1* exon 11 mutant SUM149PT breast cancer cells and their complemented derivative expressing wild-type BRCA1 (SUM149PT + BRCA1) with the PARPi olaparib for 1 h, a previously established condition that promotes ssDNA gap formation (Simoneau et al. 2021). We then monitored ssDNA gap accumulation by S1 DNA fiber analysis (Quinet et al. 2017). We pulse-labeled cells with a thymidine analog, CIdU (Fig. 1A, red), for 20 min, followed by labeling with the second thymidine analog, IdU (Fig. 1A, green), for 1 h and concomitant treatment with 10 μM olaparib. We collected cells immediately after PARPi removal (T0), as well as 30 min (T30) or 60 min (T60) after PARPi removal, and assessed for the presence of ssDNA gaps on the IdU-labeled tracts by adding the ssDNA-specific S1 nuclease (see Fig. 1A, scheme). The shorter DNA fiber tracts generated by S1 cleavage were used as a readout for the presence of ssDNA gaps (Quinet et al. 2017). We found that PARPi treatment led to ssDNA gap accumulation in both BRCA1-proficient (SUM149PT + BRCA1) and -deficient (SUM149PT) cells. However, although most of the gaps were promptly repaired within 30 min after olaparib removal in BRCA1-proficient SUM149PT + BRCA1 cells, they were not repaired in BRCA1-deficient SUM149PT cells (Fig. 1B). Similar results were also obtained by monitoring gap repair 60 min after olaparib removal, further supporting the conclusion that gaps failed to be repaired in a BRCA1-deficient background. The same kinetics of ssDNA gap repair were also observed using the *BRCA1* exon 11 mutant ovarian cancer cell line UWB1.289 (referred to here as UW) and its complemented derivative expressing wild-type BRCA1 (UW + BRCA1), confirming that the observed phenotype is not cell type-specific (Supplemental Fig. S1A). Moreover, we confirmed ssDNA gap accumulation upon PARPi treatment also in human HEK293T cells, for which we have established procedures to efficiently downregulate BRCA1 using siRNA (Supplemental Fig. S1B,C).

A limitation of the DNA fiber technique is that it only provides information on the presence or absence of gaps. Instead, EM provides an accurate measure of the gap length and informs on whether the ssDNA gaps are located at the fork junction or at internal sites (Jackson and Vindigni 2022). PARPis were previously suggested to promote the accumulation of ssDNA gaps by activating PRIMPOL repriming (Simoneau et al. 2021; Tirman et al. 2021) or by causing defects in Okazaki fragment processing (Vaitsiankova et al. 2022). Both events are expected to promote the formation of internal postreplicative gaps behind the fork junction. Thus, we initially investigated how BRCA1 status affects the frequency and size of internal daughter strand ssDNA gaps upon treatment with the PARPi olaparib using EM (Fig. 1C). We found that treatment with 10 μM olaparib for 1 h promoted the formation of internal daughter strand gaps in ∼16% of the replication intermediates in SUM149PT + BRCA1 cells, a rare event in untreated cells (Fig. 1D, cf. first and second columns). Very few ssDNA gaps remained detectable after 30 min, suggesting that most of the gaps were quickly repaired after PARPi removal in SUM149PT + BRCA1 cells (Fig. 1D, second and third columns), in agreement with the DNA fiber data (Fig. 1B). The absence of BRCA1 caused a slight increase in the frequency of replication intermediates containing ssDNA gaps at T0 compared with BRCA1-proficient cells (Fig. 1D, second and fifth columns). Notably, the frequency of internal ssDNA gaps did not change between the T0 and T30 time points in BRCA1-deficient cells, indicating that the gaps could not be repaired when BRCA1 was missing (Fig. 1D, fifth and sixth columns).

Next, we measured the size of the internal gaps by monitoring local changes in filament thickness (Jackson and Vindigni 2022). The internal gaps in PARPi-treated SUM149PT + BRCA1 cells had a median length of 120 nt at T0 (Fig. 1E, second column). In stark contrast, the median length of the ssDNA gaps detected at T0 increased to 177 nt in BRCA1-deficient cells, suggesting that ssDNA gaps are likely resected by nucleases in the absence of BRCA1 (Fig. 1E, fifth column), as already proposed in previous studies (Tirman et al. 2021; Hale et al. 2023; García-Rodríguez et al. 2024). Furthermore, the gap size in BRCA1-deficient cells slightly increased at T30, suggesting that although most of the resection activity occurred during the time frame of olaparib treatment, some limited resection might have continued after olaparib removal (Fig. 1E, fifth and sixth columns). In contrast to the internal ssDNA gaps, the median length of junction gaps only increased by ∼15 nt in BRCA1-deficient cells compared with BRCA1-proficient cells, suggesting that junction gaps might not be effectively processed by nucleases, at least under our experimental conditions (Supplemental Fig. S1D,E).

### MRE11 activity regulates daughter strand ssDNA gap repair but not their formation

**Figure 2.**
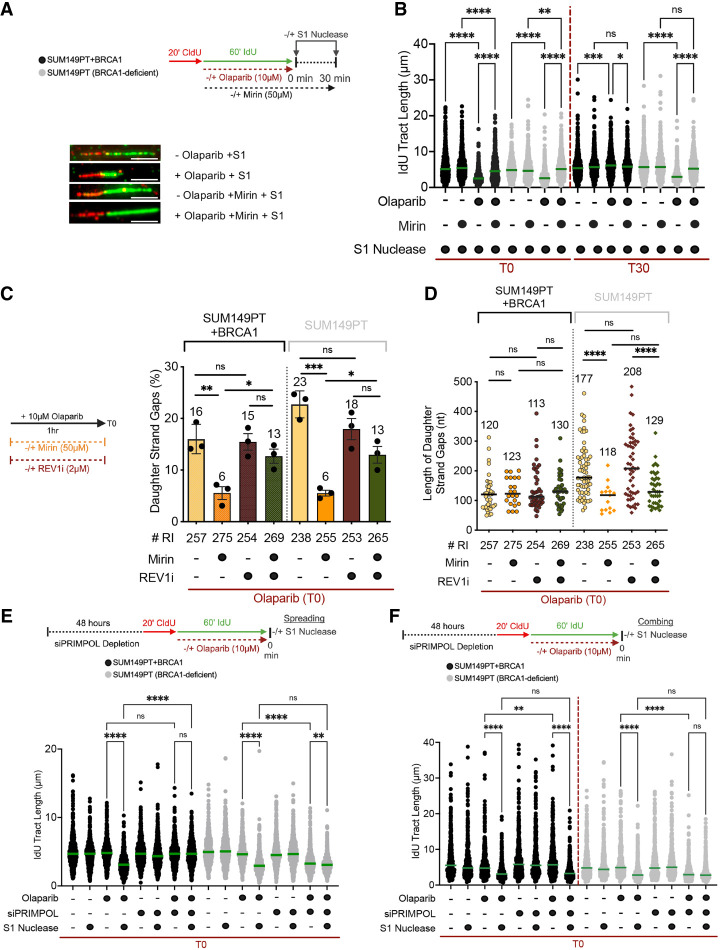
MRE11 inhibition rescues ssDNA gap repair without affecting ssDNA gap formation (see also Supplemental Fig. S2). (*A*, *top*) Schematic of the DNA fiber spreading assay with the S1 nuclease in the presence and absence of Mirin. (*Bottom*) Representative images of DNA fibers in SUM149PT treated with S1 ± 10 μM olaparib for 1 h and ±50 μM Mirin. Scale bar, 10 μm. (*B*) Dot plot and median of IdU tract lengths in SUM149PT and SUM149PT + BRCA1 cells treated with S1 ± 10 μM olaparib and ±50 μM Mirin for 1 h. The S1 nuclease was added immediately after (time 0) and 30 min (time 30) after olaparib removal (*n* = 3). At least 180 tracts were scored for each sample. Statistics: Kruskal–Wallis followed by Dunn's multiple comparisons test. (ns) Nonsignificant, (*) *P* < 0.0332, (**) *P* < 0.0021, (***) *P* < 0.0002, (****) *P* < 0.0001. (*C*, *left*) Schematic of electron microscopy experiment in the presence and absence of Mirin and REV1i (JH-RE-06). (*Right*) Percentage of replication forks with daughter strand gaps in SUM149PT + BRCA1 and SUM149PT cells treated with 10 μM olaparib ± 50 μM Mirin and ±2 μM REV1i (JH-RE-06) for 1 h. Cells were collected immediately after PARPi removal (T0). The first and fifth columns are repeated data from Figure 1D used for easier comparison between samples in different figures. “# RI” indicates the number of analyzed replication intermediates. (*n* = 3). Columns indicate mean ± SD. Mean values are shown *above* each data set. Statistics: unpaired *t*-test. (ns) Nonsignificant, (*) *P* < 0.0332, (**) *P* < 0.0021, (***) *P* < 0.0002. (*D*) Length of daughter strand ssDNA gaps in nucleotides in SUM149PT and SUM149PT + BRCA1 cells treated as in *C*. The first and fifth columns are repeated data from Figure 1D used for easier comparison between samples in different figures. “# RI” indicates the number of analyzed replication intermediates. (*n* = 3). Statistics: unpaired *t*-test with Welch correction. (ns) Nonsignificant, (****) *P* < 0.0001. Horizontal bars indicate median. Median values are shown *above* each data set. (*E*, *top*) Schematic of the DNA fiber assay performed by using the spreading technique with the S1 nuclease in the presence and absence of PRIMPOL. (*Bottom*) Dot plot and median of IdU tract lengths in SUM149PT and SUM149PT + BRCA1 cells treated with S1 ± 10 μM olaparib and ±siPRIMPOL. The S1 nuclease was added immediately after olaparib removal (time 0) (*n* = 3). (*F*, *top*) Schematic of the DNA fiber assay performed by using the combing technique with the S1 nuclease in the presence and absence of PRIMPOL. (*Bottom*) Dot plot and median of IdU tract lengths in SUM149PT and SUM149PT + BRCA1 cells treated with S1 ± 10 μM olaparib and ±siPRIMPOL. The S1 nuclease was added immediately after olaparib removal (time 0) (*n* = 3). At least 130 tracts were scored for each sample in *E* and *F*. Statistics in *E* and *F*: Kruskal–Wallis followed by Dunn's multiple comparisons test. (ns) Nonsignificant, (**) *P* < 0.0021, (****) *P* < 0.0001.

Previous studies suggested that MRE11 plays a key role in ssDNA gap resection (Hashimoto et al. 2010; Kolinjivadi et al. 2017; Tirman et al. 2021; Hale et al. 2023; García-Rodríguez et al. 2024). Using the S1 DNA fiber approach, we found that addition of the MRE11 inhibitor Mirin concomitant with olaparib treatment fully restored ssDNA gap repair in BRCA1-deficient cells within 30 min after olaparib removal, suggesting that inhibiting MRE11 nuclease activity re-established efficient gap repair (Fig. 2A,B). Interestingly, addition of Mirin partially suppressed ssDNA accumulation already at T0 (immediately after olaparib removal) in both BRCA1-proficient and -deficient cells, indicating that inhibiting MRE11 activity altered the kinetics of ssDNA gap repair independently of BRCA status (Fig. 2B). Analogous results were obtained in the UW and UW + BRCA1 cells, as well as in HEK293T cells, confirming that the effect of Mirin on ssDNA gap repair is not cell type-specific (Supplemental Fig. S1A–C). The partial suppression of ssDNA gap accumulation by MRE11 inhibition at T0 could be due to either accelerated gap repair or reduced gap formation. To distinguish between these two possibilities, we performed an additional experiment using the REV1 inhibitor JH-RE-06 (Wojtaszek et al. 2019) on the basis of previous results showing that REV1 is required for the repair of ssDNA gaps (Nayak et al. 2020; Taglialatela et al. 2021). Indeed, we found that JH-RE-06 addition re-established ssDNA gaps even in cells treated with Mirin (Supplemental Fig. S2A), indicating that MRE11 is not responsible for gap formation. Next, we validated the same conclusions using EM (Fig. 2C,D). In agreement with the DNA fiber data, we found that addition of Mirin concomitant with olaparib treatment significantly reduced the frequency of ssDNA gaps in both BRCA1-proficient and -deficient cells (Fig. 2C). Moreover, addition of Mirin reduced the size of the ssDNA gaps in BRCA1-deficient cells, supporting the model that MRE11 overresects ssDNA gaps in a BRCA1-deficient background (Fig. 2D). Conversely, addition of Mirin did not significantly affect the size of the ssDNA gaps in BRCA1-proficient cells, suggesting that MRE11 activity is restricted in these cells. This restriction results in more limited processing of the gaps, which may be below the detection threshold of the EM technique. We also found that addition of JH-RE-06 restored ssDNA gap accumulation in Mirin-treated cells, confirming that blocking MRE11 activity promotes ssDNA gap repair without compromising gap formation (Fig. 2C). Collectively, our data support previous models in which MRE11 overresects internal ssDNA gaps in BRCA1-deficient cells. In addition, they show that inhibiting MRE11 activity rescues the ability of BRCA1-deficient cells to repair internal ssDNA gaps without affecting their initial formation.

### PRIMPOL repriming drives the formation of a subset of ssDNA gaps

We next sought to investigate the mechanisms that promote the initial formation of internal daughter strand ssDNA gaps upon PARPi treatment. First, we performed new S1 DNA fiber assays in PRIMPOL-depleted cells to test whether PRIMPOL repriming is required for this process, as previously suggested (Simoneau et al. 2021). Indeed, we found that depletion of PRIMPOL rescued the IdU tract-shortening phenotype caused by addition of the S1 nuclease in the SUM149PT + BRCA1 cells, confirming that PRIMPOL repriming is an important driver of ssDNA gap formation in PARPi-treated cells (Fig. 2E, fourth and eighth columns; Supplemental Fig. S2B). However, we recently showed that the DNA spreading technique does not resolve sister chromatids, preventing the detection of strand-specific alterations such as ssDNA gaps (Supplemental Fig. S2C; Meroni et al. 2024). This observation left open the possibility that the effect of the S1 nuclease might not be detectable in the PRIMPOL-depleted cells, as ssDNA gaps present on only one of the two strands would not be identifiable with the spreading technique. To rule out this possibility, we repeated the S1 assay using the DNA combing technique, which unlike the spreading technique can detect strand-specific alterations (Fig. 2F, fourth and eighth columns). When using DNA combing, we found that the IdU-labeled tract lengths were no longer rescued by the depletion of PRIMPOL. This indicates that PRIMPOL repriming is not the only pathway promoting ssDNA formation in PARPi-treated cells.

Next, we extended the analysis to the SUM149PT BRCA1-deficient cells. When using the DNA spreading approach, we found that addition of the S1 nuclease caused a marked reduction in the length of the IdU tracts, as in the BRCA1-proficient cells (Fig. 2E, 11th and 12th columns). Conversely, the length of the IdU-labeled tracts was only slightly reduced by the S1 nuclease when PRIMPOL was depleted (Fig. 2E, 15th and 16th columns). However, the interpretation of the results with the PRIMPOL-depleted cells is complicated by the fact that, differently from the BRCA1-proficient cells, loss of PRIMPOL alone caused a significant shortening of the IdU-labeled tracts even in the absence of S1 in the BRCA1-deficient SUM149PT cells (Fig. 2E, 11th and 15th columns). Similar results were confirmed using PRIMPOL knockout SUM149PT cells (Ramakrishnan et al. 2024), indicating that the observed phenotype is not due to an off-target effect of the PRIMPOL siRNA (Supplemental Fig. S2D,E). We next repeated the same analysis by DNA combing. Similar to the results obtained using the spreading technique, the length of the IdU-labeled tracts detected by combing was not significantly affected by addition of S1 when PRIMPOL was depleted (Fig. 2F, 15th and 16th columns). The same results were recapitulated with the PRIMPOL knockout cells (Supplemental Fig. S2F). The significant shortening associated with the loss of PRIMPOL alone in PARPi-treated BRCA1-deficient cells suggests that PRIMPOL repriming might play a more important role in facilitating replication fork progression in PARPi-treated BRCA1-deficient cells relative to the BRCA1-proficient cells. At the same time, this significant shortening might hinder the detection of any additional decreases in tract length associated with the addition of S1 nuclease. Shorter fibers could fall below the threshold limit of detection, hindering the capacity to accurately evaluate the contribution of PRIMPOL repriming in ssDNA gap formation in BRCA1-deficient cells. Collectively, these data indicate that although PRIMPOL repriming is a key driver of ssDNA gap formation in both BRCA1-proficient and -deficient PARPi-treated cells, an alternative pathway may also contribute to the formation of ssDNA gaps on one of the two chromatids. This alternative pathway seems to be more readily detectable in BRCA1-proficient cells compared with BRCA1-deficient cells. Interestingly, recent studies suggested that PARPis cause a defect in Okazaki fragment processing (Vaitsiankova et al. 2022). Combined with these studies, our results suggest that there is a subset of gaps that still form in the absence of PRIMPOL on the opposite chromatid, likely because of defective Okazaki fragment processing. Importantly, the results previously obtained upon MRE11 and/or REV1 inhibition using the spreading technique could be reproduced using the combing technique (Supplemental Fig. S2G). Because the combing technique can distinguish strand-specific alterations, these results suggest that ssDNA gaps are processed in the same manner by MRE11 and REV1 regardless of whether they form on the leading or lagging strand of a DNA replication fork.

### MRE11 uses distinct mechanisms to resect ssDNA gaps versus DSBs

**Figure 3.**
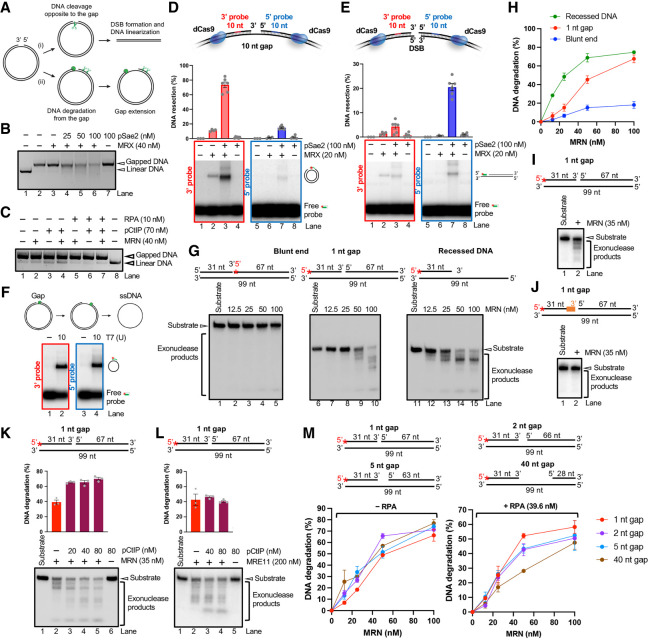
The MRE11 complex extends DNA gaps through CtIP-stimulated exonuclease activity (see also Supplemental Fig. S3). (*A*) Schematic of the plasmid-based DNA substrate with a 10 nt long DNA gap, indicating various degradation scenarios. See the text for details. (*B*) Nuclease assays with a 10 nt long gapped DNA substrate with MRX and increasing concentrations of phosphorylated Sae2 (pSae2). (Lane *1*) The linearized substrate is included for reference. Shown is a representative gel from three independent experiments. (*C*) Nuclease assays with a 68 nt long gapped DNA substrate with MRN, phosphorylated CtIP (pCtIP), and RPA. (Lane *8*) The linearized substrate is included for reference. Shown is a representative gel from two independent experiments. (*D*) Annealing resection assays with a 10 nt long gapped DNA substrate and MRX and pSae2, as indicated. (*Top*) Zoomed-in view of the ssDNA gap, indicating the positions of the probes used to detect DNA resection. (*Middle*) Quantitation of resection efficiency measured with the 3′-specific probe (red; *left*) or the 5′-specific probe (blue; *right*). Averages are shown; *n* ≧ 3; error bars indicate SEM. (*Bottom*) Representative gels from at least three independent experiments. (*E*) Annealing resection assays with a linearized DNA substrate (otherwise identical to *D*) and MRX and pSae2, as indicated. (*Top*) Zoomed-in view of the DSB, indicating the positions of the probes used to detect DNA resection. (*Middle*) Quantitation of resection efficiency measured with the 3′-specific probe (red; *left*) or the 5′-specific probe (blue; *right*). Averages are shown; *n* ≧ 3; error bars indicate SEM. (*Bottom*) Representative gels from at least three independent experiments. (*F*) Annealing resection assays of the gapped plasmid-based DNA substrate with the T7 exonuclease. Shown is a representative gel from three independent experiments showing that both probes exhibit a similar annealing efficacy. (*G*) Exonuclease assays with the indicated oligonucleotide-based DNA substrates and increasing concentrations of MRN, as indicated. (*Top*) Cartoons of the various DNA substrates. The red asterisk represents the position of the ^32^P label. (*Bottom*) Representative gels from three independent experiments. (*H*) Quantitation of experiments shown in *G*. Averages are shown; *n* = 3; error bars indicate SEM. (*I*) Exonuclease assays with 1 nt long gapped DNA substrate and MRN, as indicated. (*Top*) A cartoon of the substrate. The red asterisk represents the position of the ^32^P label. (*Bottom*) Representative gel from three independent experiments. (*J*) Exonuclease assays as in *I* but with a substrate containing eight phosphorothioate bonds at the 3′ side of the gap, represented by the orange line in the cartoon. (*Top*) A cartoon of the substrate. The red asterisk represents the position of the ^32^P label. (*Bottom*) Representative gel from three independent experiments. (*K*) Exonuclease assays with a 1 nt long gapped DNA substrate, MRN, and increasing concentrations of pCtIP, as indicated. (*Top*) A cartoon of the substrate. The red asterisk represents the position of the ^32^P label. (*Middle*) Quantitation of DNA degradation. Averages are shown; *n* = 3; error bars indicate SEM. (*Bottom*) Representative gel from three independent experiments. (*L*) Exonuclease assays with a 1 nt long gapped DNA substrate, MRE11, and increasing concentrations of pCtIP, as indicated. (*Top*) A cartoon of the substrate. The red asterisk represents the position of the ^32^P label. (*Middle*) Quantitation of DNA degradation. Averages are shown; *n* = 3; error bars indicate SEM. (*Bottom*) Representative gel from three independent experiments. (*M*) Quantitation of exonuclease assays with substrates having gaps of different lengths, with MRN, in the absence or presence of human RPA, as shown in Supplemental Figure S3F. Averages are shown; *n* = 3; error bars indicate SEM.

To obtain mechanistic insights into the activity of the MRE11 complex at DNA gaps, we next used in vitro reconstitution biochemistry with purified recombinant proteins (Supplemental Fig. S3A). We incubated a plasmid-based DNA substrate with a 10 nt long gap (Fig. 3A) with the MRE11 complex. We opted to first use the yeast MRX complex (Mre11–Rad50–Xrs2) together with its cofactor, phosphorylated Sae2 (pSae2), which displays a higher activity in vitro compared with the human MRE11–RAD50–NBS1 (MRN) complex with phosphorylated CtIP (pCtIP) homologs. We envisioned two possibilities. If the MRX complex cleaves the DNA strand opposite the gap via its ssDNA endonuclease activity (Trujillo and Sung 2001; Deshpande et al. 2016), the DNA substrate would be linearized (Fig. 3A, scenario i). Alternatively, if the MRE11 complex extends the gap, progressively smaller reaction intermediates would be visible as a smear below the substrate band (Fig. 3A, scenario ii), resulting from either a 3′–5′ exonuclease activity or endonucleolytic incisions of the 5′-terminated gapped DNA strand. The activity of MRX without or with pSae2 did not lead to detectable DNA linearization and instead resulted in gap extension (Fig. 3B; Supplemental Fig. S3A). On the other hand, MRX–pSae2 exhibited a very efficient DNA endonuclease activity near the ends of a linear 2.5 kb long dsDNA substrate (Supplemental Fig. S3B), in agreement with previous studies (Cannavo and Cejka 2014; Cannavo et al. 2019). Furthermore, although MRX can incise DNA opposite a nick within plasmid-based DNA, this activity is very weak in comparison and is not stimulated by pSae2 (Supplemental Fig. S3C). Using a plasmid with a 68 nt long gap, we similarly observed an inefficient DNA linearization by human MRN independent of pCtIP (Fig. 3C). Importantly, DNA linearization was largely eliminated in the presence of RPA, arguing that ssDNA cleavage at gaps, away from DSBs, is suppressed under physiological conditions when RPA is present. Collectively, these data suggest that the MRE11 complex does not efficiently cleave the ssDNA region of gaps but rather extends the gap, supporting scenario ii in Figure 3A.

We next investigated whether the MRX–pSae2 complex extended the gap by using its 3′–5′ exonuclease activity or endonucleolytic DNA cutting expected to target the 5′-terminated DNA strand (Cannavo and Cejka 2014). We first performed the nuclease assay with the recombinant proteins and then incubated the reaction products with two distinct radioactive probes that are complementary to regions on either side of the gap at a distance of 10 bp within the adjacent dsDNA. If resection occurred, the probes would anneal to the exposed DNA (Fig. 3D). Strikingly, we observed a signal indicative of preferential degradation of the 3′-terminated DNA strand at gaps (Fig. 3D). As a control, we introduced a DSB into the same location instead of the DNA gap. In this case, the 5′ DNA strand was degraded (Fig. 3E), in agreement with previous data showing that MRE11 primarily targets the 5′ strand in the context of DNA end resection (Cannavo and Cejka 2014; Reginato et al. 2017; Cannavo et al. 2019). In both substrates, we placed catalytically inactive Cas9 (dCas9), which binds 90 bp away from the gap at the 3′ end side and 160 bp away from the gap at the 5′ end side, to prevent exonucleolytic degradation from proceeding further away from the gap (Fig. 3D,E). We also confirmed that both probes annealed to ssDNA with similar efficacy, as seen upon complete degradation of the gapped DNA strand by the T7 exonuclease (Fig. 3F). Notably, the MRE11 complex was previously shown to endonucleolytically incise DNA past protein blocks and at secondary structures bound by RPA near DSBs (Wang et al. 2017), which facilitated resection of protein-blocked and harpin-capped DNA breaks (Lobachev et al. 2002). However, away from DSBs, the endonuclease activity of the MRE11 complex was suppressed (Reginato et al. 2024). Based on our new results, we conclude that at DNA gaps away from DSBs, the MRX–pSae2 ensemble preferentially extends gapped DNA in the 3′–5′ direction, presumably using its exonuclease activity, in contrast to its activity at DSBs that are degraded in the opposite 5′–3′ direction (Fig. 3D,E).

To further validate our observations with the human MRE11 complex, we turned to oligonucleotide-based DNA substrates. First, we compared DNA degradation by the MRN (MRE11–RAD50–NBS1) exonuclease starting from a blunt end, a 1 nt long gap, or a recessed DNA end (Fig. 3G,H; Supplemental Fig. S3A). MRN displayed a preference for the 1 nt long gap and the recessed DNA end, while DNA degradation starting from the blunt end was inefficient (Fig. 3G,H). We observed a similar preference with human MRE11 (without RAD50 and NBS1) (Supplemental Fig. S3A,D,E). The introduction of phosphorothioate linkages on the 3′ side of the gap abrogated the DNA degradation by MRN (Fig. 3I,J), confirming that the observed gap extension was due to the exonuclease activity of the complex starting from the gap rather than the endonuclease activity of MRN initiating at the DSB. The MRN exonuclease activity from the 1 nt long gap was stimulated by phosphorylated CtIP (pCtIP) (Fig. 3K), similar to yeast pSae2 stimulating the exonuclease activity of MRX (Cannavo et al. 2019; Tamai et al. 2024). This stimulation by pCtIP was not observed when MRN was replaced with MRE11 (Fig. 3L), in agreement with similar data from yeast (Cannavo et al. 2019). We found that MRN acted similarly on DNA gaps of different lengths (1, 2, 5 and 40 nt long) (Fig. 3M; Supplemental Fig. S3A,F). When RPA was added to the reaction, the extension of longer DNA gaps was moderately inhibited, possibly resulting from MRN and RPA competing for access to the DNA substrate (Fig. 3M; Supplemental Fig. S3A,F). A similar effect was observed using MRE11 alone in both the absence and presence of RPA (Supplemental Fig. S3A,G,H). Together, our data establish that the MRE11 complex has an evolutionarily conserved ability to extend DNA gaps through its 3′–5′ exonuclease activity, which is stimulated by pSae2/pCtIP, and that unlike at DNA ends, the endonuclease activity of the MRE11 complex is inefficient at cleaving the 5′-terminated strand flanking the gap or the ssDNA itself.

### EXO1 and DNA2 extend DNA gaps in the 5′–3′ direction

**Figure 4.**
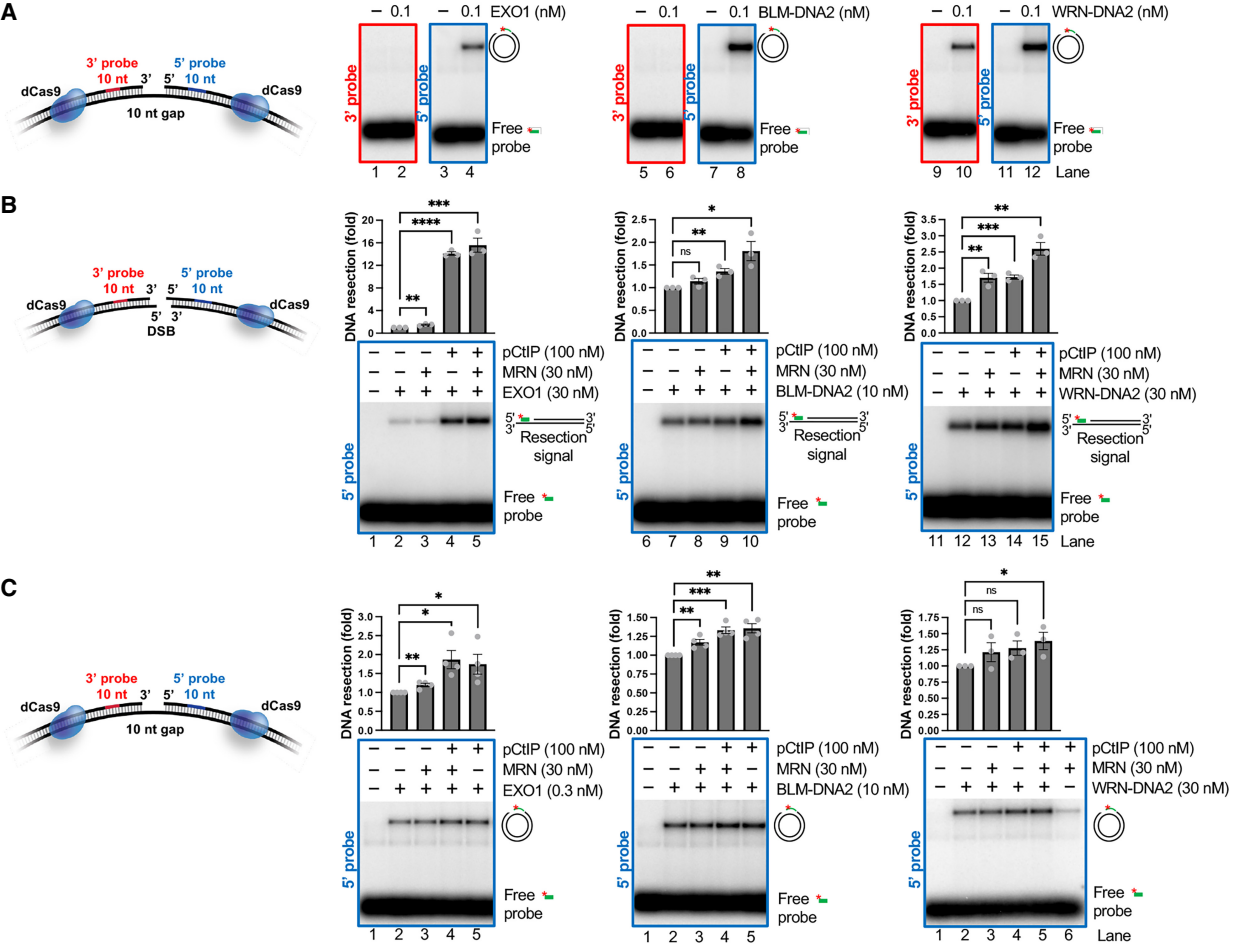
EXO1 and DNA2 extend DNA gaps in the 5′–3′ direction (see also Supplemental Fig. S4). (*A*) Annealing DNA end resection assays with the 10 nt long gapped plasmid-based DNA substrate and EXO1, BLM–DNA2, or WRN–DNA2, as indicated. All samples contained 267.7 nM RPA. The signal resulting from the degradation in the 3′ (red; *left*) and 5′ (blue; *right*) directions is presented. Representative gels from three independent experiments are shown. (*B*) Annealing DNA end resection assays with the linear plasmid-based DNA substrate and EXO1, BLM–DNA2, or WRN–DNA2 without or with MRN and pCtIP. All samples contained 267.7 nM RPA. (*Top*) Quantitation of resection efficiency measured with the 5′-specific probe. Averages are shown; *n* = 3; error bars indicate SEM. Statistics: unpaired *t*-test. (ns) Nonsignificant, (*) *P* < 0.0332, (**) *P* < 0.0021, (***) *P* < 0.0002, (****) *P* < 0.0001. (*Bottom*) Representative gels from three independent experiments. (*C*) Annealing DNA end resection assays with the 10 nt long gapped plasmid-based DNA substrate and EXO1, BLM–DNA2, or WRN–DNA2 without or with MRN and pCtIP. All samples contained 267.7 nM RPA. (*Top*) Quantitation of resection efficiency measured with the 5′-specific probe. Averages are shown; *n* = 3; error bars indicate SEM. Statistics: unpaired *t*-test. (ns) Nonsignificant, (*) *P* < 0.0332, (**) *P* < 0.0021, (***) *P* < 0.0002. (*Bottom*) Representative gels from three independent experiments.

EXO1 and DNA2 have also been implicated in ssDNA gap resection (Hale et al. 2023; García-Rodríguez et al. 2024; Nusawardhana et al. 2024). In addition, the BLM and WRN helicases were previously shown to stimulate the end resection activity of DNA2 (Nimonkar et al. 2011; Sturzenegger et al. 2014). To study the mechanism by which these long-range resection nucleases process ssDNA gaps, we incubated the 10 nt long gapped DNA substrate with EXO1 alone or DNA2 together with either the BLM or WRN helicases (Supplemental Fig. S3A). As above, we immobilized catalytically inactive Cas9 (dCas9) on the dsDNA flanking the gap to prevent the complete degradation of the substrate. All enzyme combinations extended the gap in the 5′–3′ direction (Fig. 4A). When WRN was tested, a weaker 3′ signal was also observed, likely due to its 3′–5′ exonuclease activity (Fig. 4A, lane 10). The short-range resection pathway components were shown to stimulate long-range resection enzymes at DSBs through a combination of structural (nonenzymatic; i.e., recruitment) and enzymatic (i.e., DNA-melting) functions (Cejka et al. 2010; Nicolette et al. 2010; Niu et al. 2010; Shim et al. 2010; Nimonkar et al. 2011; Cannavo et al. 2013; Myler et al. 2017; Reginato et al. 2017; Yang et al. 2017; Gobbini et al. 2018). Using matched DNA substrates with either a DSB or a gap within the same DNA sequence, we were able to recapitulate the stimulation of EXO1, BLM–DNA2, and WRN–DNA2 by MRN–pCtIP at DNA ends (Fig. 4B). However, under the same experimental conditions, MRN–pCtIP was comparatively inefficient in stimulating the long-range resection nucleases at DNA gaps (Fig. 4C; Supplemental Fig. S4A,B). We note that EXO1 and DNA–WRN degraded plasmid-length nicked DNA with the same efficacy as gapped DNA (Supplemental Fig. S4C–F), suggesting that any potential initial resection of short gaps by MRN is unlikely to strongly promote EXO1/DNA2 activity. Finally, MRN–pCtIP also did not promote resection of nicked DNA by EXO/WRN–DNA2 (Supplemental Fig. S4G,H). Together, these data point to another important difference between the previously described mechanism of DSB resection and the mechanism of ssDNA gap resection (Fig. 4C; Supplemental Fig. S4A,B). These observations are consistent with the model in which short- and long-range resection enzymes act on opposite ends of DNA gaps.

### CtIP, EXO1, DNA2, WRN, and BLM cooperate with MRE11 in ssDNA gap resection in PARPi-treated cells

**Figure 5.**
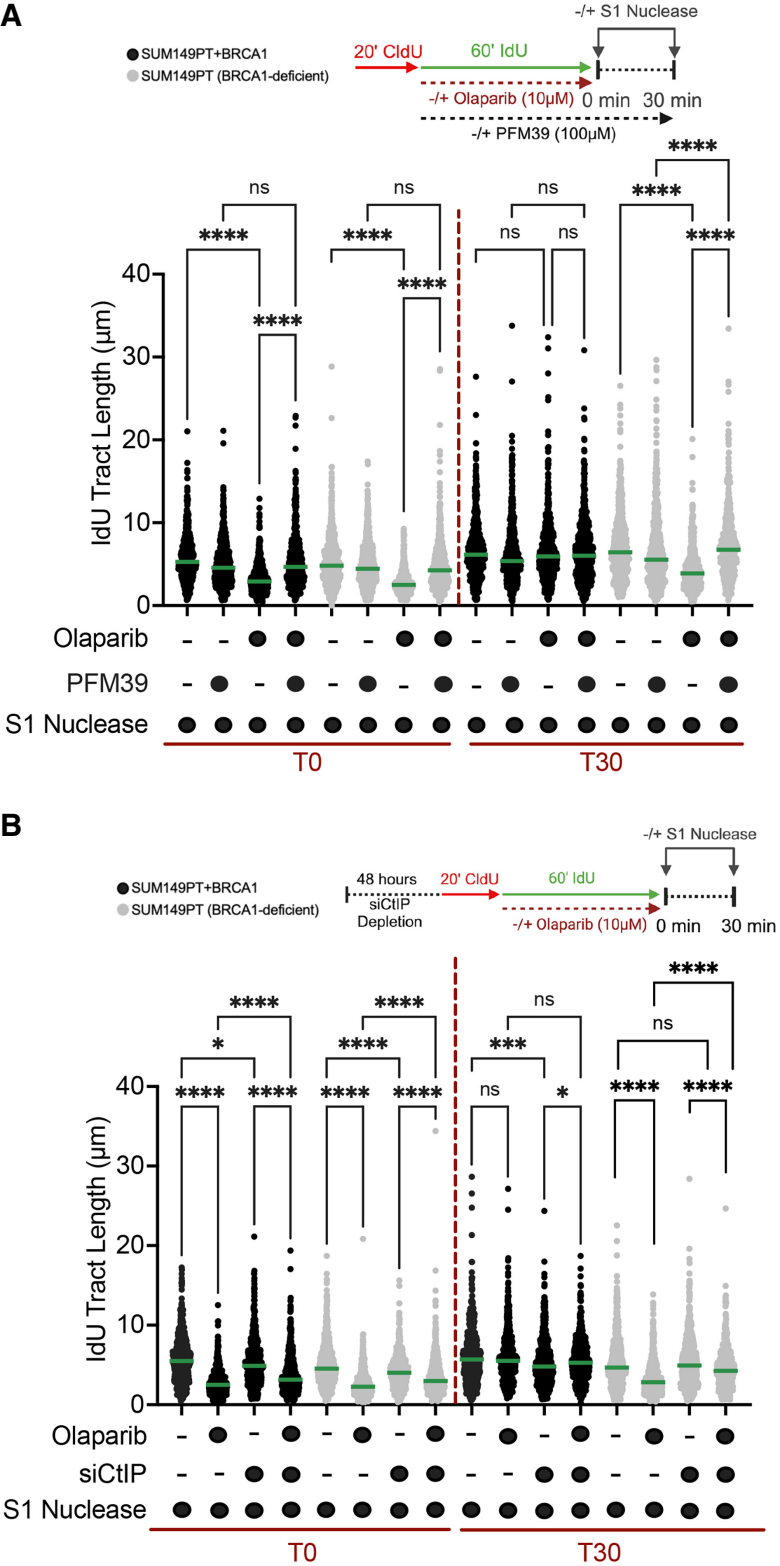
Inhibition of MRE11 3′–5′ exonuclease activity and loss of CtIP rescue ssDNA gap repair in PARPi-treated cells (see also Supplemental Fig. S5). (*A*, *top*) Schematic of the DNA fiber spreading assay with the S1 nuclease in the presence and absence of PFM39. (*Bottom*) Dot plot and median of IdU tract lengths in SUM149PT and SUM149PT + BRCA1 cells treated with S1 ± 10 μM olaparib for 1 h and ±100 μM PFM39. The S1 nuclease was added immediately after (time 0) and 30 min (time 30) after olaparib removal (*n* = 3). (*B*, *top*) Schematic of the DNA fiber spreading assay with the S1 nuclease in the presence and absence of CtIP. (*Bottom*) Dot plot and median of IdU tract lengths in SUM149PT and SUM149PT + BRCA1 cells treated with the S1 nuclease ± 10 μM olaparib for 1 h and ±siCtIP. The S1 nuclease was added immediately after (time 0) and 30 min (time 30) after olaparib removal (*n* = 3). At least 170 tracts were scored for each sample in *A* and *B*. Statistics in *A* and *B*: Kruskal–Wallis followed by Dunn's multiple comparisons test. (ns) Nonsignificant, (*) *P* < 0.0332, (***) *P* < 0.0002, (****) *P* < 0.0001.

**Figure 6.**
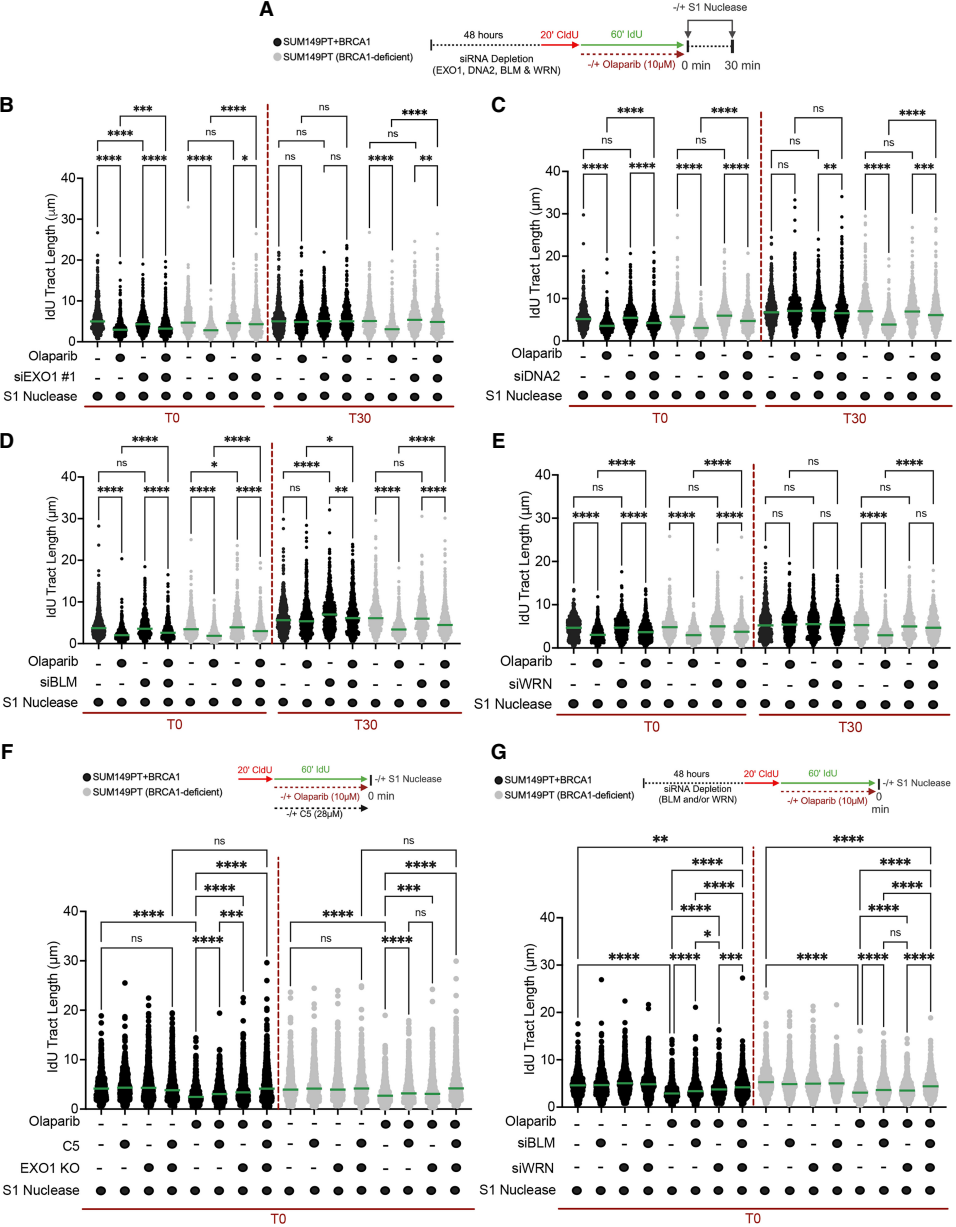
EXO1, DNA2, BLM, and WRN regulate ssDNA gap repair in PARPi-treated cells (see also Supplemental Fig. S6). (*A*) Schematic of the DNA fiber spreading assay with the S1 nuclease in the presence and absence of EXO1, DNA2, WRN, and BLM. (*B*) Dot plot and median of IdU tract lengths in SUM149PT and SUM149PT + BRCA1 cells treated with S1 ± 10 μM olaparib for 1 h and ±siEXO1 #1. The S1 nuclease was added immediately after (time 0) and 30 min (time 30) after olaparib removal (*n* = 3). (*C*) Dot plot and median of IdU tract lengths in SUM149PT and SUM149PT + BRCA1 cells treated with S1 ± 10 μM olaparib for 1 h and ±siDNA2. The S1 nuclease was added immediately after (time 0) and 30 min (time 30) after olaparib removal (*n* = 3). (*D*) Dot plot and median of IdU tract lengths in SUM149PT and SUM149PT + BRCA1 cells treated with S1 ± 10 μM olaparib for 1 h and ±siBLM. The S1 nuclease was added immediately after (time 0) and 30 min (time 30) after olaparib removal (*n* = 3). (*E*) Dot plot and median of IdU tract lengths in SUM149PT and SUM149PT + BRCA1 cells treated with S1 ± 10 μM olaparib for 1 h and ±siWRN. The S1 nuclease was added immediately after (time 0) and 30 min (time 30) after olaparib removal (*n* = 3). (*F*) Dot plot and median of IdU tract lengths in SUM149PT and SUM149PT + BRCA1 control sgAAVS1 or pooled sgEXO1 KO cells treated with S1 ± 10 μM olaparib for 1 h and ±C5 inhibitor. (*G*) Dot plot and median of IdU tract lengths in SUM149PT and SUM149PT + BRCA1 cells treated with S1 ± 10 μM olaparib for 1 h and ±siWRN and siBLM. At least 110 tracts were scored for each sample in *B*–*G*. Statistics in *B*–*G*: Kruskal–Wallis followed by Dunn's multiple comparisons test. (ns) Nonsignificant, (*) *P* < 0.0332, (**) *P* < 0.0021, (***) *P* < 0.0002, (****) *P* < 0.0001.

We sought to validate the conclusions of the biochemical data in cells treated with PARPis. To test the contribution of the exonuclease and endonuclease activity in ssDNA gap resection, we first tested two small molecule derivatives of the MRE11 inhibitor Mirin, termed PFM03 and PFM39, which were previously shown to selectively target either the 5′–3′ endonuclease or the 3′–5′ exonuclease activity of MRE11, respectively (Shibata et al. 2014). Using the S1 DNA fiber approach, we found that like Mirin, treatment with PFM39 fully suppressed ssDNA accumulation immediately after PARPi removal (T0) in both BRCA1-proficient and -deficient cells, supporting the model in which the 3′–5′ exonuclease activity of MRE11 is required for ssDNA gap resection (Fig. 5A). Similar results were obtained by monitoring ssDNA gaps 30 min after PARPi removal, where the ssDNA gaps that persisted in the BRCA1-deficient cells were suppressed by PFM39 treatment (Fig. 5A). Conversely, treatment with two different concentrations of the PFM03 inhibitor did not affect ssDNA gap accumulation, suggesting that the 5′–3′ endonuclease activity of MRE11 might not be required for gap processing, in agreement with the biochemical data (Supplemental Fig. S5A–C). However, an important limitation of these experiments is that, differently from PFM39, the PFM03 inhibitor is toxic to the cells, complicating a proper interpretation of the results (Supplemental Fig. S5D,E). Based on the biochemical data showing that the MRN exonuclease activity was stimulated by CtIP, we also tested the contribution of CtIP in gap resection in PARPi cells by depleting CtIP by siRNA. In agreement with the biochemical data, the S1 DNA fiber experiments confirmed that CtIP depletion partially suppressed ssDNA gap accumulation at both the T0 and T30 time points (Fig. 5B; Supplemental Fig. S5F). We also observed a similar partial suppression in ssDNA gap accumulation upon depletion of the long-range resection nucleases EXO1 and DNA2, as well as WRN and BLM, which was again consistent with the biochemical results (Fig. 6A–E; Supplemental Fig. S6A–E). Analogous results were obtained using the chemical inhibitor of DNA2, C5 (Supplemental Fig. S6F). Notably, we found that the combination of EXO1 loss and DNA2 inhibition fully restored ssDNA gap repair (Fig. 6F), suggesting that resection at the 5′ end occurs through either the EXO1- or DNA2-dependent pathway. Similarly, combined depletion of WRN and BLM led to a more significant rescue in gap repair compared with the depletion of the individual proteins (Fig. 6G; Supplemental Fig. S6H). Combined with the biochemical data, the DNA fiber studies support a model in which MRE11 resects gaps in the 3′–5′ direction by virtue of its exonuclease activity, whereas EXO1 and DNA2–WRN (or DNA2–BLM) resect the opposite side of the ssDNA gap in a 5′–3′ directionality, resulting in bidirectional gap resection. Moreover, these data argue that suppressing the activity of either MRE11 on the 3′ end of the gap or EXO1 and DNA/WRN (or DNA2/BLM) on the 5′ end is sufficient to re-establish gap repair.

### ssDNA gaps persist in PARPi-treated cells until the following round of the cell cycle

**Figure 7.**
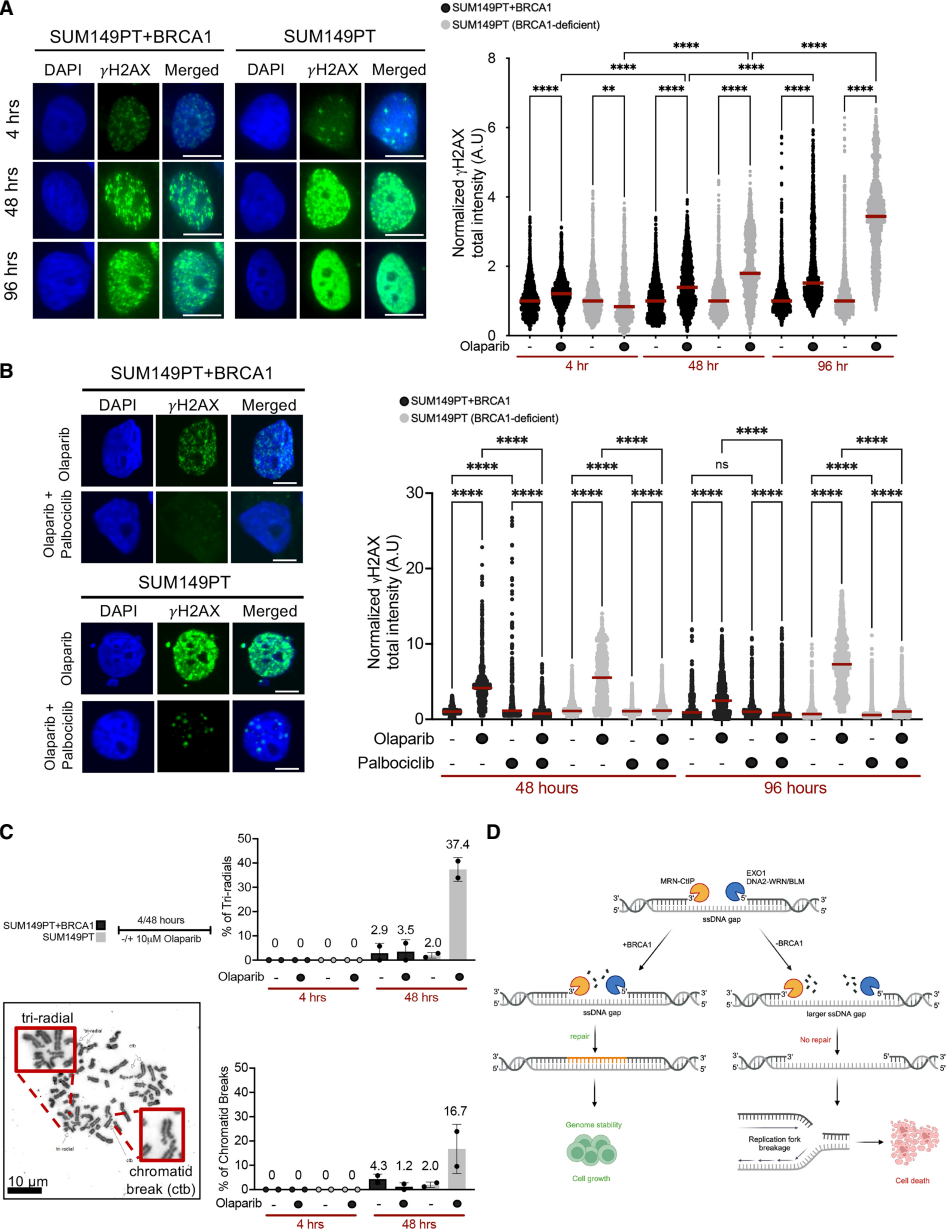
DNA breaks only form after prolonged PARPi treatment (see also Supplemental Fig. S7). (*A*, *left*) Representative immunofluorescence images of γ-H2AX foci in SUM149PT and SUM149PT + BRCA1 cells treated with 10 μM olaparib for the indicated durations. Scale bar, 11 μm. (*Right*) Dot plot of normalized γ-H2AX focus intensity (A.U). At least 300 cells were analyzed per condition per biological replicate (*n* = 3). Statistics: Kruskal–Wallis followed by Dunn's multiple comparisons test. (**) *P* < 0.0021, (****) *P* < 0.0001. (*B*, *left*) Representative immunofluorescence images of γ-H2AX foci in SUM149PT and SUM149PT + BRCA1 cells treated with 10 μM olaparib plus 10 μM palbociclib for the indicated durations. Scale bar, 11 μm. (*Right*) Dot plot of normalized γ-H2AX focus intensity (A.U). At least 300 cells were analyzed per condition per biological replicate (*n* = 3). Statistics: Kruskal–Wallis followed by Dunn's multiple comparisons test. (ns) Nonsignificant, (****) *P* < 0.0001. (*C*, *left*) Schematic (*top*) and representative image (*bottom*) of metaphase spread experiment. Red boxes indicate representative images of triradial and chromatid breaks (ctb). (*Right*) The percentage of triradials (*top*) and chromatid breaks (*bottom*) in SUM149PT + BRCA1 and SUM149PT cells ±10 μM olaparib for 4 and 48 h. (*D*) Working model for the mechanism of ssDNA gap resection. MRN–CtIP resect ssDNA gaps in the 3′–5′ direction, whereas EXO1 and DNA2/WRN/BLM operate in the 5′–3′ direction. ssDNA gaps are overresected in BRCA1-deficient cells, preventing their repair. The unrepaired ssDNA gaps collide with ongoing replication forks, leading to fork breakage and promoting cell death in the absence of BRCA1.

To further support the notion that MRE11 endonuclease activity does not cleave ssDNA gaps, we measured the accumulation of DNA breaks by γ-H2AX focus analysis. Indeed, we found that treatment with 10 μM olaparib for 4 h induced very low levels of DNA breaks (Fig. 7A). However, significantly higher levels of DNA breaks were detected at later time points—48 and 96 h after olaparib treatment. Moreover, BRCA1-deficient cells accumulated more breaks compared with BRCA1-proficient cells, and this phenotype was further exacerbated 96 h after olaparib treatment. Similar results were obtained when monitoring the γ-H2AX by flow cytometry (Supplemental Fig. S7A). Combined with the biochemical data, these results suggest that the ssDNA gaps that form in PARPi-treated cells are not actively cleaved by nucleases during their initial processing but may be converted into DNA breaks at later stages. To test whether entry into the S phase during the following round of the cell cycle is important for DNA damage accumulation, we arrested cells in the second G1 with the CDK4/6 inhibitor palbociclib. Addition of palbociclib did not cause any significant perturbation in the cell cycle at 18 h of treatment when cells are still in the first phase of the cell cycle (Supplemental Fig. S7B). However, cells were arrested in the second G1 at 48 h after palbociclib treatment (Supplemental Fig. S7C), in agreement with previous findings (Simoneau et al. 2021). At 48 and 96 h after olaparib treatment, the palbociclib-treated cells displayed a significantly reduced level of DNA breaks compared with the untreated cells (Fig. 7B). These findings support a model in which ssDNA gaps persist in PARPi-treated cells throughout the following round of the cell cycle and are eventually converted into DSBs upon collision with an incoming replication fork in the second S phase, as suggested in recent studies (Simoneau et al. 2021). Importantly, our observation that the levels of breaks are higher in BRCA1-deficient cells compared with BRCA1-proficient cells is also consistent with the model in which the broken forks arising from collisions between ssDNA gaps and replication forks cannot be effectively repaired in a BRCA1-deficient background. In agreement with this model, karyotyping of these cells showed that treatment with PARPis for 48 h led to a marked accumulation of broken metaphases, including increased levels of chromatid breaks and triradials, only in a BRCA1-deficient background (Fig. 7C).

## Discussion

Various replication stress-inducing agents promote the accumulation of postreplicative ssDNA gaps on the daughter strands of DNA replication forks. These gaps need to be promptly processed and repaired to ensure genome stability. Here, we combined single-molecule DNA fiber analysis, EM, and biochemical methods to study the mechanism by which nucleases resect ssDNA gaps in cancer cells treated with PARPis. Our findings highlight how MRN–CtIP, EXO1, and DNA2–WRN/BLM process ssDNA gaps bidirectionally through a mechanism that is distinct from their previously described function at DNA ends and does not involve single-stranded DNA cleavage (Fig. 7D). Using EM, we also found that these nucleases preferentially process internal ssDNA gaps relative to gaps that are present at the fork junction. Moreover, our DNA fiber data show that the processing of ssDNA gaps by nucleases alters the kinetics of gap repair in both BRCA1-proficient and -deficient cells. Excessive resection of the internal gaps in BRCA1-deficient cells leads to larger gaps that cannot be repaired and are eventually converted into a DNA break when cells enter the S phase of the next cell cycle.

PARPi treatment promotes the accumulation of ssDNA gaps (Cong et al. 2021; Simoneau et al. 2021). These gaps must be quickly repaired to ensure genome stability (Tirman et al. 2021). However, gap repair is impaired in BRCA1-deficient cells, leading to an aberrant accumulation of ssDNA gaps and promoting PARPi sensitivity. Our work sheds light on how the structure and nuclease-mediated processing of ssDNA gaps affect their ability to be repaired in BRCA1-deficient cells. Previous EM studies showed the MRE11 nuclease promotes the resection of internal ssDNA gaps in *Xenopus* extracts depleted for another central homologous recombination factor, RAD51 (Hashimoto et al. 2010), and that MRE11 activity selectively targeted internal ssDNA gaps but not gaps present at the fork junctions in BRCA2- or RAD51-depleted extracts (Hashimoto et al. 2010; Kolinjivadi et al. 2017). However, these studies were performed in extracts that were either untreated or treated with methylmethanesulfonate (MMS), raising the question of whether the same conclusions apply to cancer cells treated with different replication stress-inducing agents. Using a combination of single-molecule DNA fiber and EM approaches, we found that PARPis induce the formation of internal ssDNA gaps behind the replication forks in both BRCA1-proficient and -deficient cells. However, the internal ssDNA gaps that form in BRCA1-deficient cells treated with PARPis are significantly larger compared with those that form in BRCA1-proficient cells. Conversely, the size of the ssDNA gaps present at the fork junction is only modestly affected by the BRCA1 status. Inhibition of MRE11 activity reduces the size of the internal ssDNA gaps detected in BRCA1-deficient cells to the same level as in BRCA1-proficient cells and re-establishes their ability to be repaired. Combined with the previous studies in *Xenopus* extracts, our work indicates that MRE11 has an evolutionarily conserved role in processing ssDNA gaps. The extensive resection of internal ssDNA gaps by MRE11, and potentially other nucleases, in BRCA1-deficient cells leads to larger gaps, which cannot be properly repaired. An interesting avenue for future studies would be to determine why internal ssDNA gaps are more extensively processed by nucleases compared with those present at fork junctions. A possible explanation is that unlike internal gaps, the junction gaps that form on the leading strand lack a 5′ end that can be processed by long resection nucleases. Additionally, the junction gaps are likely forming because of the polymerase stalling on one or both strands and might not be readily accessible to MRE11 processing because of steric hindrance with core replisome components. Conversely, the internal gaps form behind the replication forks either because of PRIMPOL repriming or because of defective Okazaki fragment processing on the lagging strand and might be more readily accessible to nucleases. The different mechanisms that promote the formation of junction gaps relative to internal gaps could also affect the downstream mechanisms that promote their repair.

Leveraging our recent discovery that the DNA combing technique (Meroni et al. 2024), unlike the spreading technique, can detect strand-specific alterations, we also investigated whether PARPis lead to the accumulation of strand-specific ssDNA gaps. Using the spreading technique, we initially found that the ssDNA gaps are no longer detectable upon PRIMPOL depletion. However, ssDNA gaps are still detectable in PRIMPOL-depleted cells when using the combing approach, suggesting that PARPis promote ssDNA gap formation on both the leading and lagging strands of a DNA replication fork. Interestingly, previous studies suggested that PARPis cause a defect in Okazaki fragment processing (Vaitsiankova et al. 2022). Combined with these studies, our results suggest that although PRIMPOL repriming is responsible for ssDNA gap formation in PARPi-treated cells, likely on the leading strand, there is a subset of gaps that still form in the absence of PRIMPOL on one of the opposite chromatids due to defective Okazaki fragment processing. Of note, we also found that the loss of PRIMPOL causes a shortening in the DNA replication tracks in BRCA1-deficient but not in -proficient cells, opening the intriguing scenario that PRIMPOL repriming is required to facilitate fork progression in a BRCA1-deficient background.

The MRE11 enzyme is a key element of the MRE11–RAD50–NBS1 (MRN) complex, which has been extensively studied for its role in the repair of double-strand breaks (DSBs) (Lisby et al. 2004; Lamarche et al. 2010; Zhao et al. 2020). MRN exhibits both 3′–5′ exonuclease capacity and an endonuclease activity on 5′-terminated DNA strands. At DNA ends, the MRN complex first uses its endonuclease activity, stimulated by CtIP, to introduce a nick at internal positions on the 5′-terminated strands (Anand et al. 2016). This is followed by the 3′-to-5′ exonuclease activity of MRN, moving from the nick back to the end of the DNA (Trujillo and Sung 2001; Neale et al. 2005; Shibata et al. 2014; Cannavo et al. 2019). Conversely, the long-range resection nucleases EXO1 or DNA2 trim the DNA in the 5′-to-3′ direction, opposite to MRN's exonuclease activity (Shibata et al. 2014; Cejka and Symington 2021). Interestingly, recent studies indicated that the same nucleases involved in DNA end resection (namely, MRE11, EXO1, and DNA2) are also required for ssDNA gap resection (Hale et al. 2023; García-Rodríguez et al. 2024; Nusawardhana et al. 2024). However, how the MRN complex and the long resection nucleases operate at ssDNA gaps remains less understood. Using in vitro biochemical assays with recombinantly purified proteins and matched DNA substrates with either a DSB or a gap within the same sequence, we discovered that these nucleases use distinct mechanisms to resect ssDNA gaps versus DSBs. First, we found that yeast MRX and human MRN have an evolutionarily conserved ability to extend DNA gaps through their 3′–5′ exonuclease activity, which is stimulated by yeast pSae2 and human pCtIP (Sebastiano et al. 2011; Tamai et al. 2024). Differently from DNA ends, we found that MRX/MRN do not use their endonuclease activity to create a nick on the 5′ end of the gap or cleave the ssDNA. Conversely, the 5′ end of the gap is trimmed by EXO1, BLM–DNA2, and WRN–DNA2. The long-range resection activity of these nucleases at DNA gaps is not stimulated by MRN–pCtIP, pointing to another important difference between the previously described mechanism of DSB resection and the mechanism of ssDNA gap resection. However, this observation is in agreement with the model in which short-range (MRE11) and long-range (EXO1/DNA2) nuclease complexes act on opposing ends of DNA gaps. The notion that ssDNA gaps are not actively cleaved by nucleases in PARPi-treated cells is further supported by our cellular assays, where we could not find any evidence for a role of MRN endonuclease activity leading directly to DNA break accumulation after PARPi treatment. This finding contradicts recent studies suggesting that MRE11 can actively cleave ssDNA gaps (Hale et al. 2023). A possible explanation for this discrepancy could be associated with the different agents used for ssDNA gap formation, which would argue that MRE11 might process ssDNA gaps with different mechanisms depending on the drug used in the experiments. However, it should be mentioned that the previous studies did not provide biochemical data to support their conclusions that MRE11 actively cleaves ssDNA gaps, leaving open the possibility that the breaks detected in these studies might originate because of a different nuclease-independent process or because of the action of a different endonuclease that converts the gaps into a break.

Interestingly, our cellular assays show that suppressing either the activity of MRE11 on the 3′ end of the gap or of EXO1 and DNA2–WRN (or DNA2–BLM) on the 5′ end is sufficient to re-establish gap repair. This finding further supports the biochemical data indicating that the ssDNA gaps are resected bidirectionally through two independent mechanisms. In addition, they argue that limiting the resection of the gaps from either the 3′ end or the 5′ end is sufficient to activate repair. We posit that the gap repair pathway can only fill gaps of limited length due to the low efficiency of the polymerases involved in this process. This could explain why blocking resection at either the 3′ end or the 5′ end is sufficient to restore gap repair. Moreover, we found that the combined inactivation of EXO1 and DNA2 leads to a more significant rescue in ssDNA gap repair compared with the inactivation of the individual proteins. This suggests that there are two separate pathways that can independently process the 5′ end of the gap. Of note, inhibiting the activity of these nucleases not only re-establishes gap repair in BRCA1-deficient cells but also accelerates the kinetics of ssDNA gap repair in BRCA1-proficient cells because ssDNA gaps are quickly repaired even during the timing of PARPi treatment. Our previous studies, along with work done in other laboratories, reported that ssDNA gaps can be repaired by either a TS or a TLS mechanism (Branzei and Szakal 2016; Piberger et al. 2020; Tirman et al. 2021). A tantalizing scenario, which would deserve further investigation, is whether the choice between these gap repair pathways is dependent on nuclease processing. Notably, previous biochemical data showed that the gaps resulting from PRIMPOL activity can be as short as 1 nt (Bianchi et al. 2013; García-Gómez et al. 2013; Mourón et al. 2013), arguing that the ssDNA gaps might need to be enlarged in a controlled manner by MRE11 and other nucleases in order to promote an error-free TS mechanism of ssDNA gap repair. However, in the absence of nuclease processing, the short gaps that form upon repriming might be quickly filled by TLS, leading to an overall acceleration in the kinetics of gap repair at the expense of fidelity.

Finally, our finding that MRN does not cleave the ssDNA gap opened the important questions of how and whether ssDNA gaps are eventually converted into a DSB. When treating cells with PARPis, we found that DNA breakage is only detected at least 48 h after PARPi treatment. Moreover, we found that DNA breakage is suppressed when we prevent entry into the second S phase of the cell cycle. Collectively, these findings suggest that ssDNA gaps persist in PARPi-treated cells throughout the following round of the cell cycle, when they are eventually converted into DSBs upon collision with an incoming replication fork in the second S phase, in agreement with recent findings (Simoneau et al. 2021). These findings have important implications for our understanding of the mechanism of PARPi toxicity. PARPis are an emerging class of anticancer drugs that have been FDA-approved for treatment of various types of cancer. These drugs are particularly effective in cancers that already have deficiencies in DNA repair mechanisms, such as those with mutations in the *BRCA1* and *BRCA2* genes. Our finding that ssDNA gaps are only converted into breaks in the following round of the cell cycle explains previous observations that PARPis create DNA damage at a more gradual pace compared with other DNA-damaging agents or replication inhibitors, as it takes several hours to days before DNA damage caused by PARPis becomes easily observable even in rapidly dividing cells (Michelena et al. 2018; Ryu et al. 2019). An interesting question for future studies would be to determine how gaps are preserved through mitosis in order to enter the second phase of the cell cycle. Importantly, our observation that the levels of breaks are higher in BRCA1-deficient cells compared with BRCA1-proficient cells is also consistent with the model in which the broken forks arising from collisions between ssDNA gaps and replication forks cannot be effectively repaired in a BRCA-deficient background and provides additional insight into why PARPis are particularly effective in an HR-deficient background.

## Materials and methods

### Cell culture and cell lines

The *BRCA1* mutant triple-negative breast cancer cells SUM149PT and their complemented revertant derivative expressing wild-type BRCA1, SUM149PT + BRCA1 (Wang et al. 2016), were grown in 500 mL of Ham F12 media (Sigma-Aldrich N6658) with 5% FBS, 100 U/mL penicillin/100 μg/mL streptomycin, 5.35 mL of 1 M HEPES, 250 μL of 1 mg/mL human insulin (Sigma-Aldrich I9278), and 500 μL of 1 mg/mL hydrocortisone (Sigma-Aldrich H0888) at 37°C and 5% CO_2_. The *BRCA1* mutant ovarian cancer cells UWB1.289 and their complemented revertant derivative expressing wild-type BRCA1, UWB1.289 + BRCA1 (signified as UW + BRCA1), were grown in 50% RPMI media (American Type Culture Collection [ATCC] 30-2001), 50% MEGM bullet kit (Lonza CC-3150) supplemented with 3% FBS, and 100 U/mL penicillin/100 μg/mL streptomycin or +400 μg/mL G418 (Millipore Sigma G8168) in the case of UW + BRCA1 at 37°C and 5% CO_2_. The human embryonic kidney 293 cells (HEK293T) (ATCC) were grown in DMEM supplemented with 10% FBS and 100 U/mL penicillin/100 μg/mL streptomycin at 37°C and 5% CO_2_.

### Drugs and cell treatments

PARP inhibitor olaparib (PARPi; Selleck Chemicals AZD2281) was dissolved in DMSO at a 20 mM concentration stock and immediately dissolved in cell growth media to the indicated final concentrations for experimental use at 37°C and 5% CO_2_. REV1 inhibitor JH-RE-06 (REV1i; AOBIOUS Laboratories GLXC-21219) (Wojtaszek et al. 2019) was dissolved in DMSO to a stock concentration of 10 mM and diluted in growth media to the indicated final concentrations for experimental use at 37°C and 5% CO_2_. REV1i was stored in solution at −20°C. MRE11 inhibitor Mirin (Millipore Sigma M9948) was dissolved in DMSO at a 50 mM stock concentration and diluted in cell growth media to a final concentration of 50 μM at 37°C and 5% CO_2_. MRE11's specific exonuclease inhibitor PFM39 (Sigma-Aldrich SML1839) (Shibata et al. 2014) was dissolved in DMSO at a 25 mM stock concentration and diluted in cell growth media to a final concentration of 100 μM, whereas the endonuclease-specific inhibitor PFM03 (MedChemExpress HY-148078s) (Shibata et al. 2014) was dissolved in DMSO at a 25 mM stock concentration and diluted in cell growth media to a final concentration of either 25 or 75 μM at 37°C and 5% CO_2_. DNA2 inhibitor C5 (DNA2i; AOBIOUS Laboratories AOB9082) was dissolved in DMSO at a 30 mM stock concentration and diluted in cell growth media to a final concentration of 30 μM at 37°C and 5% CO_2_. CDK4/6 inhibitor palbociclib (Selleck Chemicals S4482) was dissolved in fresh DMSO at a stock concentration of 20 mM and diluted in cell growth media to a final concentration of 10 μM at 37°C and 5% CO_2_.

### S1 nuclease DNA fiber assay

Exponentially growing cells (80,000–100,000) were plated 2 days prior to treatment. Next, they were pulse-labeled for 20 min with 20 μM CldU (5-chloro-2′-deoxyuridine; Millipore Sigma C6891-100MG), followed by one wash with PBS at 37°C and then labeled for 60 min with 200 μM IdU (5-iodo-2′-deoxyuridine; Millipore Sigma I7125-5G) in the presence and absence of PARP inhibitor olaparib at a concentration of 10 μM (Selleck Chemicals S1060) followed by two washes with PBS at 37°C.

For the experiments with the MRE11, REV1, and DNA2 inhibitors, 10 μM olaparib was added during the 1 h of IdU treatment concomitantly with 50 μM MRE11 inhibitor Mirin (Millipore Sigma M9948), 2 μM REV1 inhibitor (AOBIOUS Laboratories AOB13138) (Wojtaszek et al. 2019), 100 μM MRE11 exonuclease activity inhibitor PFM39 (Sigma-Aldrich SML1839) (Shibata et al. 2014), 25 or 75 μM MRE11 endonuclease activity inhibitor PFM03 (MedChemExpress HY-148078) (Shibata et al. 2014), or 30 μM DNA2 inhibitor C5 (AOBIOUS Laboratories AOB9082). One hour after drug treatment, cells were washed with PBS at 37°C, lysed, and collected for S1 nuclease treatment (time 0). For the experiments at 30 min (time 30) or 60 min (time 60) after olaparib removal, the same inhibitors were added again to the media before lysing and S1 nuclease treatment. For experiments with HEK293T cells, cells were depleted with the BRCA1 siRNA 48 h before treatment with the thymidine analogs, and olaparib and Mirin were added before cells were lysed and collected for S1 nuclease treatment as described above. For the experiments with siRNA depletion of BLM, DNA2, CtIP, EXO1, PRIMPOL, and WRN, the siRNAs were also added 48 h prior to thymidine analog incorporation and drug treatment. The procedure for the preparation of the siRNA-depleted cells and the EXO1 knockout cells is detailed in the Supplemental Material, and the sequences of all the siRNA used in this study are listed in Supplemental Table S1. The procedures for the RT-qPCR experiments and Western analysis for the detection of depleted proteins are also detailed in the Supplemental Material.

### DNA spreading

After thymidine incorporation, exponentially growing cells were permeabilized with CSK100 (100 mM NaCl, 10 mM MOPS at pH 7, 3 mM MgCl_2_, 300 mM sucrose, 0.5% Triton X-100 in water) for 5 min before being washed once with PBS. Next, permeabilized cells were washed with S1 buffer (930 mM sodium acetate at pH 4.6, 10 mM zinc acetate, 5% glycerol, 50 mM NaCl in water) before the addition of 20 U/mL S1 nuclease (Thermo Fisher Scientific 18001-016) for 30 min at 37°C. Cells were harvested by scraping in 300 μL of PBS/0.1% BSA, pelleted, and resuspended in 30–100 μL of PBS/0.1% BSA depending on the pellet size. To spread cells on positively charged glass slides, 2 μL of cells was mixed with 6 μL of lysis buffer (200 mM Tris-HCl at pH 7.5, 50 mM EDTA, 0.5% SDS in water) at the top of slides. After 5 min of incubation of the cells and lysis buffer on slides, the slides were tilted at a 20°–45° angle to spread the fibers at a constant low rate. After 10–20 min at room temperature, dried DNA on slides was then fixed with newly prepared 3:1 methanol:glacial acetic acid mixture for 5 min. Slides were then dried on a flat surface and stored at 4°C until staining.

To immunostain the DNA fibers, DNA was rehydrated with two washes of PBS for 5 min. Next, the DNA fibers were denatured with freshly prepared 2.5 M HCl for 1 h at room temperature before being washed three times with PBS and blocked with 5% BSA for 1 h at 37°C. The fibers were stained with rat anti-BrdU (1/75; Abcam Ab6326) and mouse anti-BrdU (1/20–1/200; BD Biosciences 347580) for 1.5 h at room temperature in PBS. Slides were then washed four times for 4 min in PBS/0.1% Tween-20 before being incubated with antirat Alexa fluor 488 and antimouse Alex fluor 568 (1/75; Thermo Fisher Scientific A21470 and A21123, respectively). DNA fibers on slides were then washed four times for 4 min in PBS/0.1% Tween-20 before being put in PBS and then mounted with Prolong Gold antifade reagent (Thermo Fisher Scientific P36930) (Quinet et al. 2017, 2020; Tirman et al. 2021).

Immunofluorescent images were acquired using LAS AF software with a Leica DM4 B automated upright microscope and a K5 microscope camera (Leica) with a 63× oil immersion objective. Between 100 and 200 DNA fibers per sample were measured with the ImageJ software per experiment (National Institutes of Health [NIH], https://imagej.nih.gov/ij/) (Schneider et al. 2012). S1 nuclease DNA fiber tract length comparison was assessed by the median IdU tract length alone.

### DNA combing

For DNA combing, cells were collected by trypsinization and embedded into agarose plugs. Immediately before combing, the DNA solution was mixed 1:1 with 2× S1 buffer (60 mM sodium acetate at pH 4.6, 20 mM zinc acetate, 10% glycerol, 100 mM NaCl in water) in the presence or absence of 40 U/mL S1 nuclease (Thermo Fisher Scientific 18001-016) and incubated for 30 min at room temperature before combing. Using a Genomic Vision combing machine, DNA fibers were stretched onto precoated, silanized glass coverslips (Moore et al. 2022; Meroni et al. 2024).

The silanized coverslip preparation procedure was as follows: Glass coverslips placed in a Wash-N-Dry coverslip rack (Sigma-Aldrich Z74368) were submerged in 70 mL of 100% ethanol for 5–10 min and then dried uncovered in the fume hood for 30 min. Each side of the coverslips was then cleaned for 30 min in a UV ozone cleaner model 3 (Thermo Fisher Scientific NC2116316), followed by dehydration for 1 h in a hybridization oven set at 70°C. Next, the coverslips were submerged in 70 mL of N-heptane (Sigma-Aldrich H2198) + 1 mL silane (Sigma-Aldrich 539279) solution in a glass desiccator jar with drierite in the fume hood overnight. After overnight silane treatment, the coverslip rack was submerged in 70 mL of N-heptane and placed in a water bath sonicator for 5 min and subsequently in distilled H_2_O for 5 min in a Branson water bath sonicator (Emerson). Coverslips were dried uncovered in the fume hood, and then the coverslip rack was submerged in fresh chloroform for 5 min in a Branson water bath sonicator. After that, the coverslips were dried by gently streaming air over the coverslips until all liquid had been removed and the coverslips appeared dry. Coverslips were stored at −20°C with drierite until use.

The DNA stretched coverslips were then baked for 3 h at 60°C and stored at −20°C. For immunostaining, DNA was denatured with fresh 0.5 M NaOH and 1 M NaCl solution for 8 min at room temperature. Coverslips were washed three times with PBS; dehydrated with 70%, 90%, and 100% ethanol for 2 min each; and blocked with 10% goat serum in PBS/0.1% Tween-20 for 1 h at room temperature. DNA fibers were immunostained with rat anti-BrdU for CldU detection (1/75; Abcam ab6326) and mouse-anti-BrdU for IdU detection (1/20; BD Biosciences 347580) for 1.5 h at 37°C, washed three times with PBS/0.01% Tween-20, and then incubated with antirat Alexa fluor 488 and antimouse Alexa fluor 568 (1/100; Thermo Fisher Scientific A-21470 and A-21124, respectively) for 1 h at room temperature. After three washes with PBS/0.01% Tween-20, coverslips were mounted with Prolong Gold antifade reagent (Thermo Fisher Scientific P36930). Images were acquired with LAS AF software using a Leica DMi8 confocal microscope with a 40×/1.15 oil immersion objective. Each experiment was performed in duplicate or triplicate as indicated in the figure legends. For each biological replicate, images were taken across the whole coverslip, and at least 50–200 fibers were counted using the “multipoint” tool of ImageJ (NIH, https://imagej.nih.gov/ij/) (Schneider et al. 2012).

### Electron microscopy

For the EM analysis of replication intermediates, SUM149PT or SUM149PT + BRCA1 cells were harvested either immediately after treatment with 10 μM olaparib (Selleck Chemicals S1060) for 1 h (time 0) or 30 min after olaparib treatment (time 30). Nontreated cells were also included as control. For the experiments with MRE11 inhibitor Mirin (Millipore Sigma M9948-5 mg) and REV1 inhibitor JH-RE-06 (AOBIOUS Laboratories AOB13138), the inhibitors were added to the SUM149PT and SUM149PT + BRCA1 cells concomitantly with olaparib treatment for 1 h at the same concentrations used in the fiber assays, and cells were collected immediately after treatment. Cells were washed, and DNA was cross-linked by incubating with 10 μg/mL 4,5′,8-trimethylpsoralen (Millipore Sigma T6137) followed by a 3 min exposure to 366 nm UV light on a precooled metal block for a total of three rounds (Jackson and Vindigni 2022). Cells were lysed and genomic DNA was isolated from the nuclei by proteinase K (Life Technologies 25530-015) digestion and chloroform–isoamyl alcohol extraction. Genomic DNA was precipitated using isopropanol and digested with PvuII HF (New England Biolabs R3151S) with the appropriate buffer for 4 h at 37°C. Replication intermediates were enriched on Qiagen plasmid mini kit columns (Qiagen 12123) and concentrated using Amicon Ultra size exclusion columns (Millipore Sigma UFC510096). Samples were prepared for EM visualization by spreading the enriched, concentrated DNA on a carbon-coated grid in the presence of benzyl-dimethyl-alkylammonium chloride (Millipore Sigma B6295) as well as N,N-dimethylformamide (Millipore Sigma 227056), followed by low-angle platinum rotary shadowing. Images were obtained on a Jeol JEM-1400 electron microscope using a bottom-mounted AMT XR401 camera. Analysis was performed using ImageJ software (NIH, https://imagej.nih.gov/ij/) (Schneider et al. 2012). Criteria used for the assignment of a replication fork include the joining of three DNA fibers into a single junction, with two generally symmetrical daughter strands and a single parental strand. EM analysis allowed for distinguishing duplexed DNA, which was expected to appear as a 10 nm thick fiber after the platinum coating step necessary for EM visualization, from ssDNA, which had a reduced thickness of 5–7 nm and appeared lighter compared with that of dsDNA. Internal ssDNA gaps behind forks were scored by measuring ssDNA regions located in the daughter arms of three-way junction fork structures and excluding ssDNA discontinuities present at fork junctions. The frequency as well as length (in nucleotides) of daughter strands and junction ssDNA gaps in a sample were computed using Prism software.

### Nuclease assays

Exonuclease assays (15 μL volume) were performed in nuclease buffer containing 25 mM Tris-acetate (pH 7.5), 5 mM magnesium chloride, 3 mM manganese chloride, 1 mM DTT, 1 mM ATP, 0.25 mg/mL BSA (New England Biolabs), and 5 nM oligonucleotide-based DNA substrate (in molecules). The recombinant proteins were then added to the reactions on ice and the samples were incubated for 2 h at 37°C. Reactions were stopped by adding 0.5 μL of 0.5 M ethylenediaminetetraacetic (EDTA) and 1 μL of 14–22 mg/mL proteinase K (Roche) and incubated for 30 min at 50°C. Finally, 16.5 μL of loading buffer (5% formamide, 20 mM EDTA, bromophenol blue) was added to all samples, and the products were separated on 15% polyacrylamide denaturing urea gels (19:1 acrylamide-bisacrylamide; Bio-Rad). The gels were fixed in fixing solution (40% methanol, 10% acetic acid, 5% glycerol) for 30 min at room temperature and dried on a 3 mm chromatography paper (Whatman). The dried gels were exposed to storage phosphor screen (GE Healthcare), scanned by a Typhoon PhosphorImager (GE Healthcare FLA 9500), and quantitated using ImageJ software.

Counter-nicking assays with plasmid-based DNA substrates (15 μL volume) were performed in nuclease buffer containing 25 mM Tris-acetate (pH 7.5), 5 mM magnesium chloride, 1 mM manganese chloride, 1 mM DTT, 1 mM ATP, 0.25 mg/mL BSA (New England Biolabs), and 100 ng of plasmid-based DNA substrate. The recombinant proteins were then added to the reactions on ice and the samples were incubated for 1 h at 37°C. Reactions were stopped with 5 µL of 2% stop solution (30 mM EDTA, 2% SDS, 30% glycerol, 1 mg/mL bromophenol blue) and 1 µL of 14–22 mg/mL proteinase K (Roche) and incubated for 15 min at 37°C. The stopped reactions were separated on 1% agarose gels containing GelRed (Biotium).

Long-range DNA end resection assays (15 μL volume) were performed in reaction buffer containing 25 mM Tris-acetate (pH 7.5), 2 mM magnesium acetate, 1 mM DTT, 1 mM ATP, 0.1 mg/mL BSA (New England Biolabs), 1 mM phosphoenolpyruvate (PEP), 80 U/mL pyruvate kinase (Sigma), and 1 nM DNA substrate (in molecules). The recombinant proteins were then added to the reactions on ice and the samples were incubated for 30 min at 37°C. The reactions were stopped with 5 µL of 2% stop solution (30 mM EDTA, 2% SDS, 30% glycerol, 1 mg/mL bromophenol blue) and 1 µL of 14–22 mg/mL proteinase K (Roche) and incubated for 15 min at 37°C. The stopped reactions were separated on 1% agarose gels containing GelRed (Biotium). The preparation of the DNA substrates and recombinant proteins for the nuclease assays is detailed in the Supplemental Material. The sequences of all the oligonucleotides used in this study are listed in Supplemental Table S2.

### Annealing DNA end resection assays

Annealing DNA end resection assays (15 μL volume) were performed in reaction buffer containing 25 mM Tris-acetate (pH 7.5), 5 mM magnesium chloride, 1 mM manganese chloride, 1 mM dithiothreitol (DTT), 1 mM ATP, 0.25 mg/mL BSA (New England Biolabs), and 1 nM DNA substrate (in molecules). Where indicated, DNA substrates were first mixed with Cas9-RNPs and incubated for 10 min at 37°C. The recombinant proteins were then added to the reactions on ice and the samples were incubated for the indicated time at 37°C (with human proteins) or at 30°C (with yeast proteins). The reactions were stopped with 5 µL of 2% stop solution (30 mM EDTA, 2% SDS, 30% glycerol, 1 mg/mL bromophenol blue) and 1 µL of 14–22 mg/mL proteinase K (Roche) and incubated for 1 h at 37°C. The stopped reactions were then supplemented with 6.5 mM final magnesium acetate and threefold excess of the radioactive probe, heated for 3 min to 60°C, and slowly cooled overnight to let the probe anneal to the resected DNA. The annealing reactions were separated on 1% agarose gels and dried on DE81 chromatography paper (Whatman). The dried gels were exposed to storage phosphor screen (GE Healthcare), scanned by a Typhoon PhosphorImager (GE Healthcare FLA 9500), and quantitated using ImageJ software.

### Immunofluorescence

Immunofluorescence experiments to measure γ-H2AX focus formation in SUM149PT and SUM149PT + BRCA1 cells were performed by culturing cells on 12 mm round coverslips in 24 well plates (see also Meroni et al. 2022). Next, cells were treated with either 10 μM olaparib (Selleck Chemicals S1060) for 4, 48, and 96 h or with the same amount of DMSO contained in the olaparib solution for the “untreated” condition. For the experiments with palbociclib (PD-0332991; Selleck Chemicals S1116), 10 μM olaparib was added concomitantly with 10 μM palbociclib for 48 and 96 h or with the same amount of DMSO contained in the olaparib plus palbociclib solutions for the “untreated” condition. At each time point, cells were fixed with 4% paraformaldehyde for 10 min at room temperature, followed by washing with phosphate-buffered saline (PBS). Next, cells were permeabilized with 0.5% Triton X-100 in PBS for 10 min and washed three to four times with PBS. After blocking with BSA for 1 h at 37^o^C, coverslips were incubated with mouse anti-phospho-Histone H2A.X (Ser139; 1/1000; clone JBW301 Millipore Sigma 05-636) primary antibody for 1 h. After washing three times with PBS, coverslips were incubated with goat antimouse IgG (H + L) cross-adsorbed secondary antibody and Alexa fluor 488 (1/1000; Thermo Fisher Scientific A-11001) for 30 min at room temperature. After three additional washes with PBS, nuclei were stained with 0.05 μg/mL DAPI in PBS for 10 min at room temperature. Coverslips were washed three times with nuclease-free water, allowed to dry at room temperature, and mounted on slides with Prolong Gold antifade reagent. Images were acquired with a Leica DM4B microscope with a 10× objective. At least 300 cells per condition were analyzed using the ImageJ analysis software (RRID: SCR_003070) to measure the γ-H2AX total intensity. To avoid bias, a macro program was used to identify nuclei outlined based on DAPI staining and to consistently measure γ-H2AX total intensity only in the nuclei. At least 300 nuclei were scored per datum, and three independent experiments were performed. The procedures for the flow cytometry analysis of γ-H2AX and of the cell cycle as well as for the cell viability assays are detailed in the Supplemental Material.

### Metaphase spreads

For metaphase spread analysis, SUM149PT and SUM149PT + BRCA1 cells were treated with 10 μM olaparib (Selleck Chemicals S1060) for 4 or 48 h with the same amount of DMSO solution for the “untreated” condition. After 4 or 48 h of treatment, cells were then incubated with 1 μg/mL colcemid (Thermo Fisher Scientific15210040) for 45 min. Culture media was removed and 4 mL of Trypsin/EDTA solution was added and incubated for 10 min. After incubation, the remaining cells were harvested by scraping. Cell suspension was centrifuged at 1000 rpm for 8 min before the remaining culture media was aspirated off and cells were gently resuspended in the remaining 1 mL left over. Next, 4 mL of warmed 37°C hypotonic solution ([NaCit/KCL]: 37.4 mM KCL, 10 mM NaCit dissolved in distilled water) was added to the suspension and incubated for 12 min. To fix solution, 1 mL of 3:1 methanol:acetic acid mixture was added and gently mixed. Solution was then centrifuged at 1000 rpm to pellet cells, and the remaining solution was aspirated. More of the fixative solution of 3:1 methanol:acetic acid was added at a higher volume of 5 mL before another round of centrifugation. Cell suspension was introduced at least two more times to the hypotonic solution and series of fixative solution steps before spreading on slides.

To spread cell solution on slides, the 3:1 methanol:acetic acid mixture was added until suspension appeared translucent. Next, two drops of 15 μL of cell solution was put onto slides. Slides were then aged for 30 min in a 90°C oven. For metaphase spread analysis, the number of metaphases was counted as well as how many broken metaphases were present at each condition. Broken metaphases were analyzed for triradial and chromatid breaks and premature centromere separation. At least 85 metaphases were analyzed per experimental condition per experiment.

### Quantification and statistical analysis

Statistical analysis was performed using Prism 8 (GraphPad Software). Details of the individual statistical tests are indicated in the figure legends and results (nonsignificant [ns], *P* < 0.0332 [*], *P* < 0.0021 [**], *P* < 0.0002 [***], and *P* < 0.0001 [****]). All experiments were repeated at least three times unless otherwise noted. Statistical differences in DNA fiber tract lengths and γ-H2AX immunofluorescence foci were determined by Kruskal–Wallis followed by Dunn's multiple comparisons test. Statistical differences for the EM experiments were determined by an unpaired *t*-test for percentage of ssDNA gaps and an unpaired *t*-test with Welch correction for the size of ssDNA gaps. Cell viability statistical analysis was determined by two-way ANOVA followed by Bonferroni test. Statistical differences for biochemical experiments were determined by an unpaired *t*-test.

### Data availability

All data supporting the findings of this study are available here, in the Supplemental Material, or via Digital Commons Data@Beker, an institutional version of Mendeley Data managed by the Bernard Becker Medical Library at Washington University School of Medicine. Any additional information required to reanalyze the data reported here is available from the lead contact on request.

## Supplemental Material

Supplement 1
