# Supplementary material for: MRN–CtIP, EXO1, and DNA2–WRN/BLM act bidirectionally to process DNA gaps in PARPi-treated cells without strand cleavage
Source: Genes Dev. 2025 May 1;39(9-10):582–602. doi: 10.1101/gad.352421.124 (PMC12047661; doi:10.1101/gad.352421.124)
Supplement: Supplement 1 [file Suppemental_Data_and_Methods.pdf]

Seppa et al.

MRN-CtIP, EXO1, and DNA2-WRN/BLM act bidirectionally to process DNA gaps in PARPi-treated cells without strand cleavage.

Supplemental Material

Supplemental Figures 1-7

Supplemental Table 1-2

Supplemental Material and Methods

Seppa Fig. S1

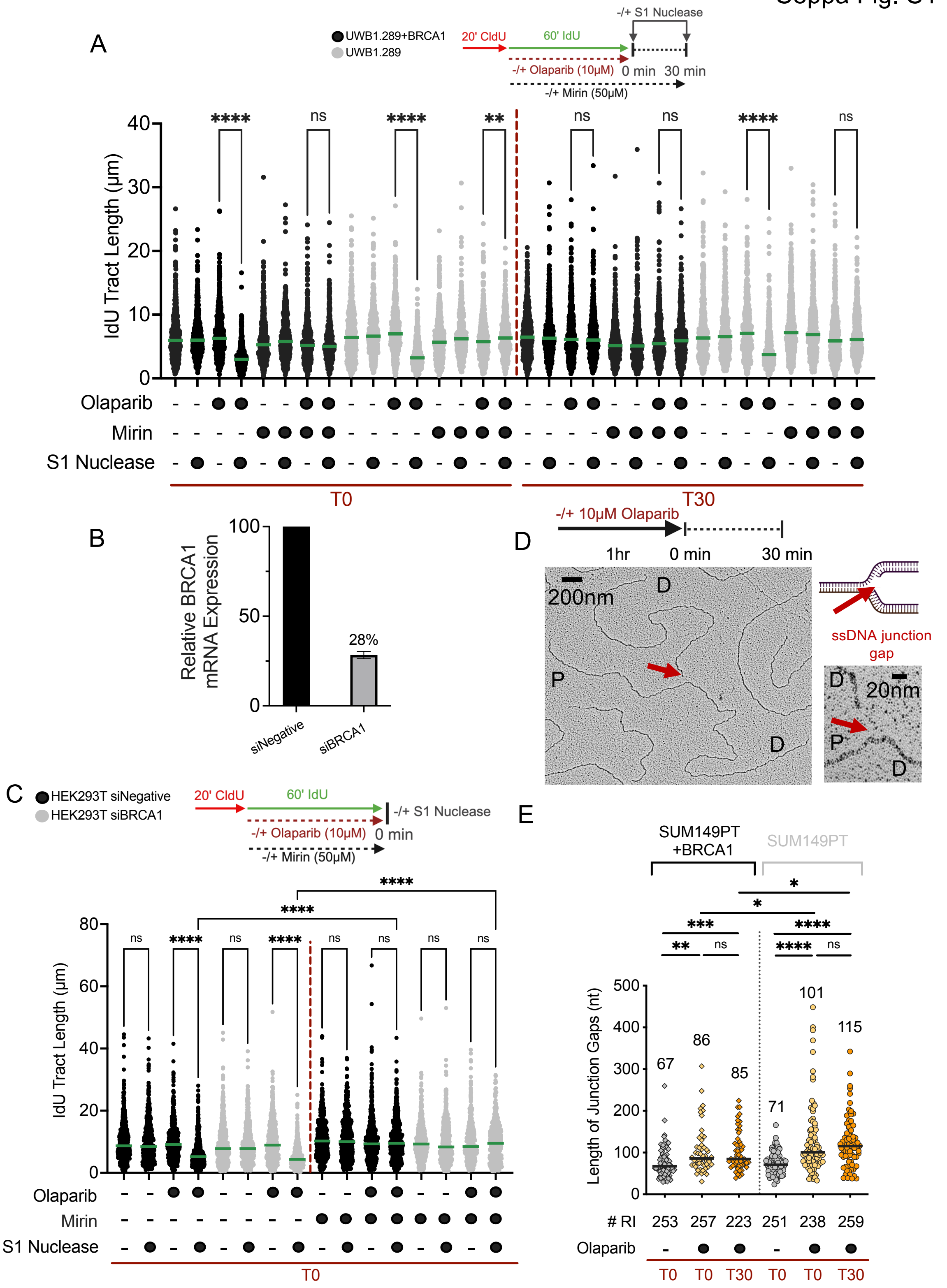

Supplemental Figure S1. ssDNA gaps cannot be repaired in BRCA1-deficient cells treated with PARPi (related to Figure 1). (A) Top, schematic of the DNA fiber spreading assay with the S1 nuclease in BRCA1-deficient UWB1.289 and BRCA1-proficient UWB1.289+BRCA1 cells.

Bottom, dot plot and median of IdU tract lengths in UWB1.289 and UWB1.289+BRCA1 cells ± 10 μM Olaparib (1 h), ± S1 nuclease, and ± 50 μM Mirin. The S1 nuclease was added immediately after (Time 0) and 30 min (Time 30) after Olaparib removal (*n*=3). At least 120 tracts were scored for each sample. Statistics: Kruskal-Wallis followed by Dunn's multiple comparisons test. *ns*, non-significant, **p < 0.0021, ****p < 0.0001. (B) mRNA expression of BRCA1 after siRNA (siNegative or siBRCA1) knockdown in HEK293T cells. (C) Top, schematic of the DNA fiber spreading assay with the S1 nuclease in HEK293T cells. Bottom, dot plot and median of IdU tract lengths in siNegative (black) and siBRCA1 (gray) HEK293T cells ± 10 μM Olaparib (1 h), ± S1 nuclease, and ± 50 μM Mirin. The S1 nuclease was added immediately after Olaparib removal (T=0) (*n*=3). At least 180 tracts were scored for each sample. Statistics: Kruskal-Wallis followed by Dunn's multiple comparisons test. *ns*, non-significant, ****p < 0.0001. (D) Top, schematic of the electron microscopy experiments of Figure S1E. Bottom left, representative electron micrograph of a replication fork containing a ssDNA gap at the fork junction. Right, magnified image of the fork junction and schematic representation of a replication fork with a junction gap. Red arrows indicate junction ssDNA gaps. P: parental strand, D: daughter strand. (E) Length of the junction ssDNA gaps in nucleotides (nt) in SUM149PT and SUM149PT+BRCA1 cells treated as in D. "# RI" indicates the number of analyzed replication intermediates. (*n*=3). Statistics: unpaired t test with Welch correction; *ns*, non-significant, *p < 0.0332, **p < 0.0021, ***p < 0.0002, ****p < 0.0001. Horizontal bars indicate median. Median values are shown on top of each data set.

Seppa Fig. S2

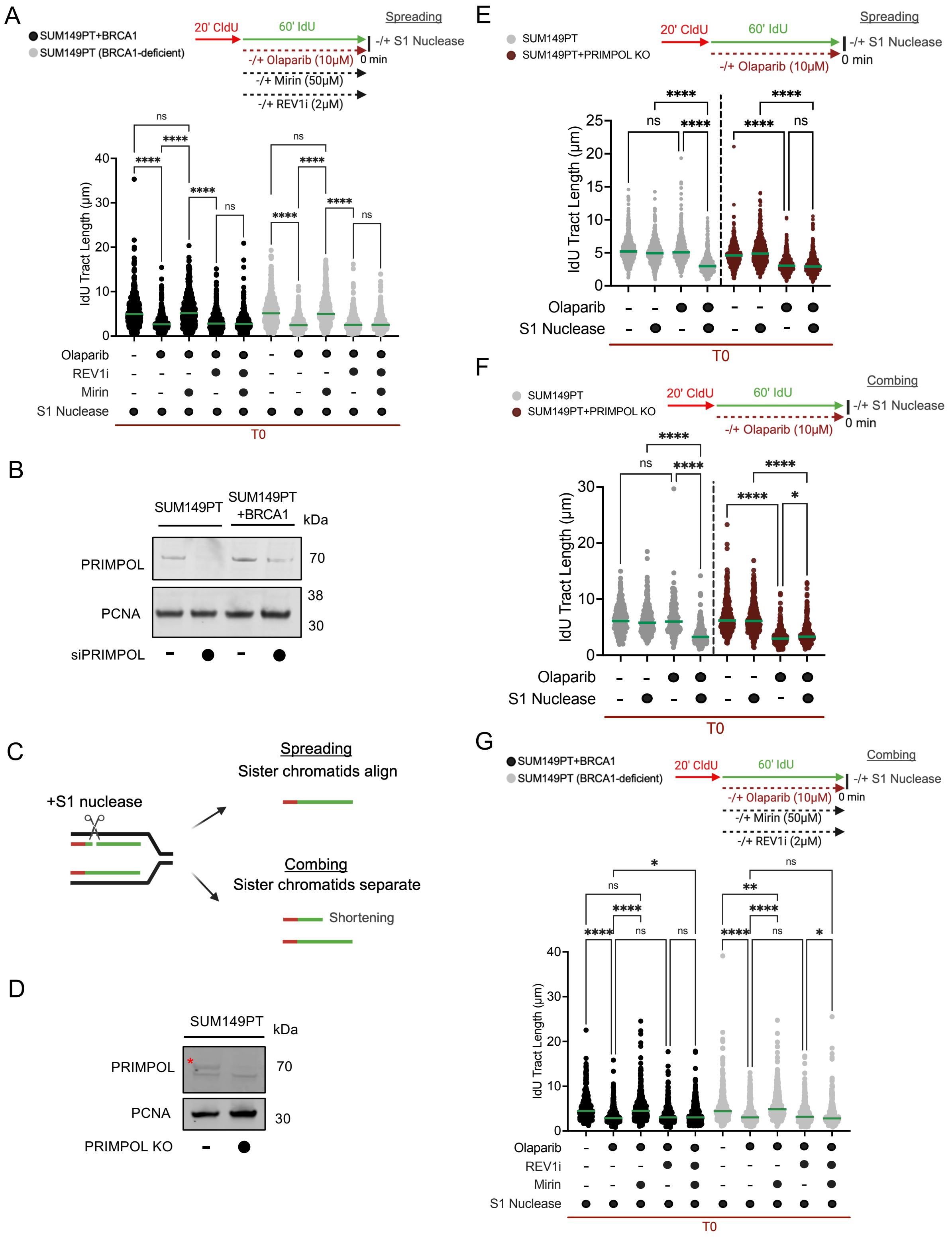

Supplemental Figure S2. MRE11 inhibition rescues ssDNA gap repair, without affecting ssDNA gap formation (related to Figure 2). (A) Top, schematic of the DNA fiber spreading assay with the S1 nuclease in the presence and absence of Mirin and REV1i (JH-RE-06). Bottom,

dot plot and median of IdU tract lengths in SUM149PT and SUM149PT+BRCA1 cells treated with 10 μM Olaparib ± 50 μM Mirin and ± 2 μM REV1i (JH-RE-06) for 1 h. The S1 nuclease was added immediately after Olaparib removal (Time 0). (*n*=3). At least 150 tracts were scored for each sample. Statistics: Kruskal-Wallis followed by Dunn's multiple comparisons test. *ns*, non-significant, ****p < 0.0001. (B) Western blot of SUM149PT and SUM149PT+BRCA1 cells depleted for PRIMPOL. (C) Schematic depicting the outcome of the spreading *versus* combing technique when ssDNA gaps are present on one sister chromatid. (D) Western blot of SUM149PT PRIMPOL KO cells. Asterisk denotes specific PRIMPOL band. (E) Top, schematic of the DNA fiber assay performed by using the spreading technique. Bottom, dot plot and median of IdU tract lengths in SUM149PT and SUM149PT PRIMPOL KO cells treated with the S1 nuclease ± 10 μM Olaparib. The S1 nuclease was added immediately after (Time 0) after Olaparib removal (*n*=3). At least 180 tracts were scored for each sample. Statistics: Kruskal-Wallis followed by Dunn's multiple comparisons test. *ns*, non-significant, ****p < 0.0001. (F) Top, schematic of the DNA fiber assay performed by using the combing technique. Bottom, dot plot and median of IdU tract lengths in SUM149PT and SUM149PT PRIMPOL KO cells treated with the S1 nuclease ± 10 μM Olaparib. The S1 nuclease was added immediately after (Time 0) after Olaparib removal (*n*=2). At least 50 tracts were scored for each sample. Statistics: Kruskal-Wallis followed by Dunn's multiple comparisons test. *ns*, non-significant, *p < 0.0332, ****p < 0.0001. (G) Top, schematic of the DNA fiber assay performed by using the combing technique. Bottom, dot plot and median of IdU tract lengths in SUM149PT and SUM149PT+BRCA1 cells treated as in A. At least 130 tracts were scored for each sample. Statistics: Kruskal-Wallis followed by Dunn's multiple comparisons test. *ns*, non-significant, *p < 0.0332, **p < 0.0021, ****p < 0.0001.

Seppa Fig. S3

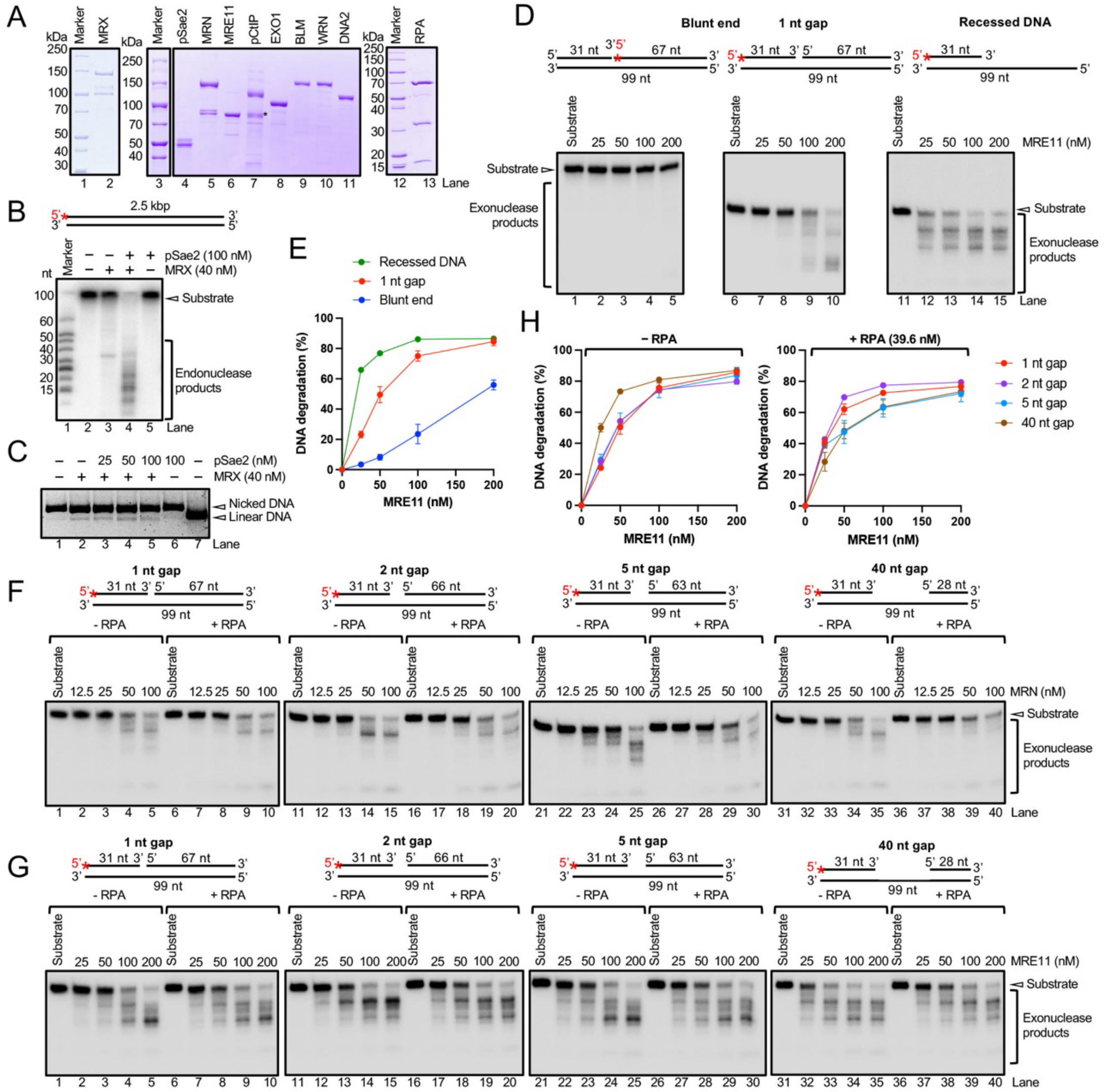

Supplemental Figure S3: The MRE11 complex extends DNA gaps through CtIP-stimulated exonuclease activity (related to Figure 3). (A) Recombinant proteins used in this study. The gel was stained with Coomassie Brilliant Blue. (B) Nuclease assays with a 5'-labeled 2.5 kbp-long linear dsDNA with MRX and phosphorylated Sae2 (pSae2), as indicated. Top, cartoon of the substrate. The red asterisk represents the position of the ^{32}P label. Shown is a representative gel from three independent experiments. (C) Nuclease assays with a nicked DNA substrate with MRX and increasing concentration of phosphorylated Sae2 (pSae2). The linearized substrates (lane 7) is shown for reference. Shown is a representative gel from three independent experiments. (D) Exonuclease assays with the indicated DNA substrates and increasing concentrations of MRE11. Top, cartoons of the substrates. The red asterisks represent the position of the ^{32}P label. Bottom, representative gels from three independent experiments. (E) Quantitation of experiments such as shown in (D). Averages shown, n = 3; error bars, SEM. (F) Exonuclease assays with gapped DNA substrate of different lengths and MRN in the absence or presence of human RPA. Top, cartoons of the substrate. The red asterisk represents the position of the ^{32}P label. Bottom, representative gels from three independent experiments. (G) Representative gels from three independent

experiments as in (F), but with MRE11 instead of MRN. (H) Quantitation of exonuclease assays such as shown in (G). Averages shown, n = 3; error bars, SEM.

Seppa Fig. S4

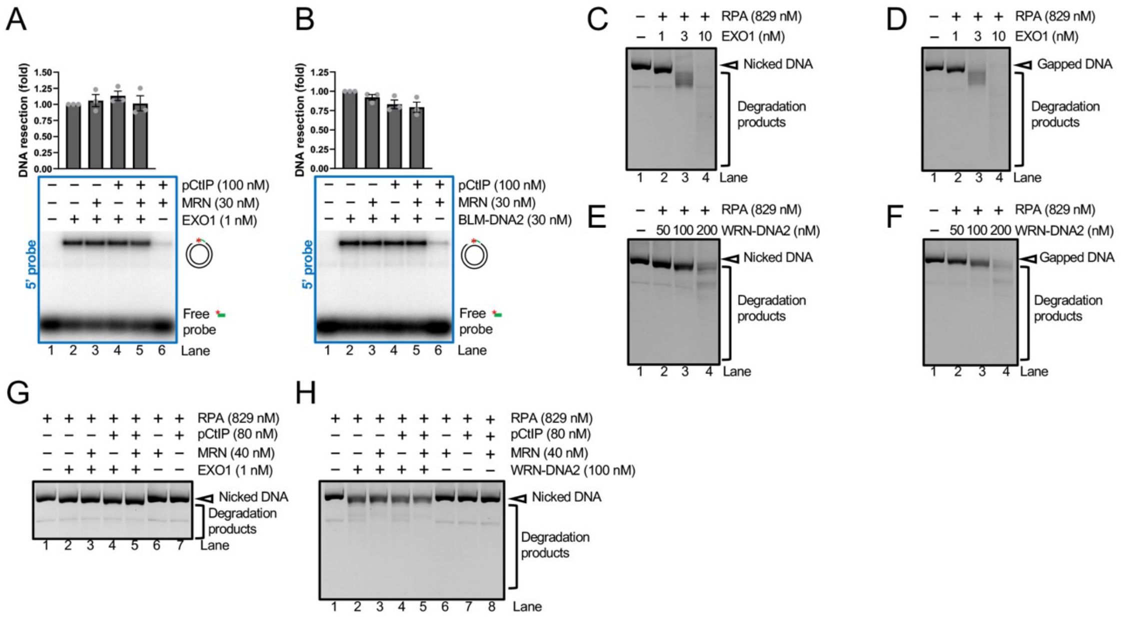

Supplemental Figure S4: DNA gaps are extended directionally by the DNA end resection enzymes (related to Figure 4). (A,B) Annealing DNA end resection assays with the 10 nt-long gapped plasmid-based DNA substrate with a high concentration of EXO1 (A) or BLM-DNA2 (B) without or with MRN and pCtIP. All samples contained 267.7 nM RPA. Top, quantitation of resection efficiency measured with the 5'-specific probe. Averages shown, n = 3; error bars, SEM. Bottom, representative gels from three independent experiments. (C,D) Nuclease assays with the nicked (C) or the 10 nt-long gapped (D) plasmid-based DNA substrate with increasing concentration of EXO1 and RPA. Shown is a representative gel from two independent experiments. (E, F) Nuclease assays with the nicked (E) or the 10 nt-long gapped (F) plasmid-based DNA substrate with increasing concentration of WRN-DNA2 and RPA. Shown is a representative gel from two independent experiments. (G) Nuclease assays with the nicked plasmid-based DNA substrate with EXO1 and RPA without or with MRN and pCtIP. Shown is a representative gel from two independent experiments. (H) Nuclease assays with the nicked plasmid-based DNA substrate with WRN-DNA2 and RPA without or with MRN and pCtIP. Shown is a representative gel from four independent experiments.

Seppa Fig. S5

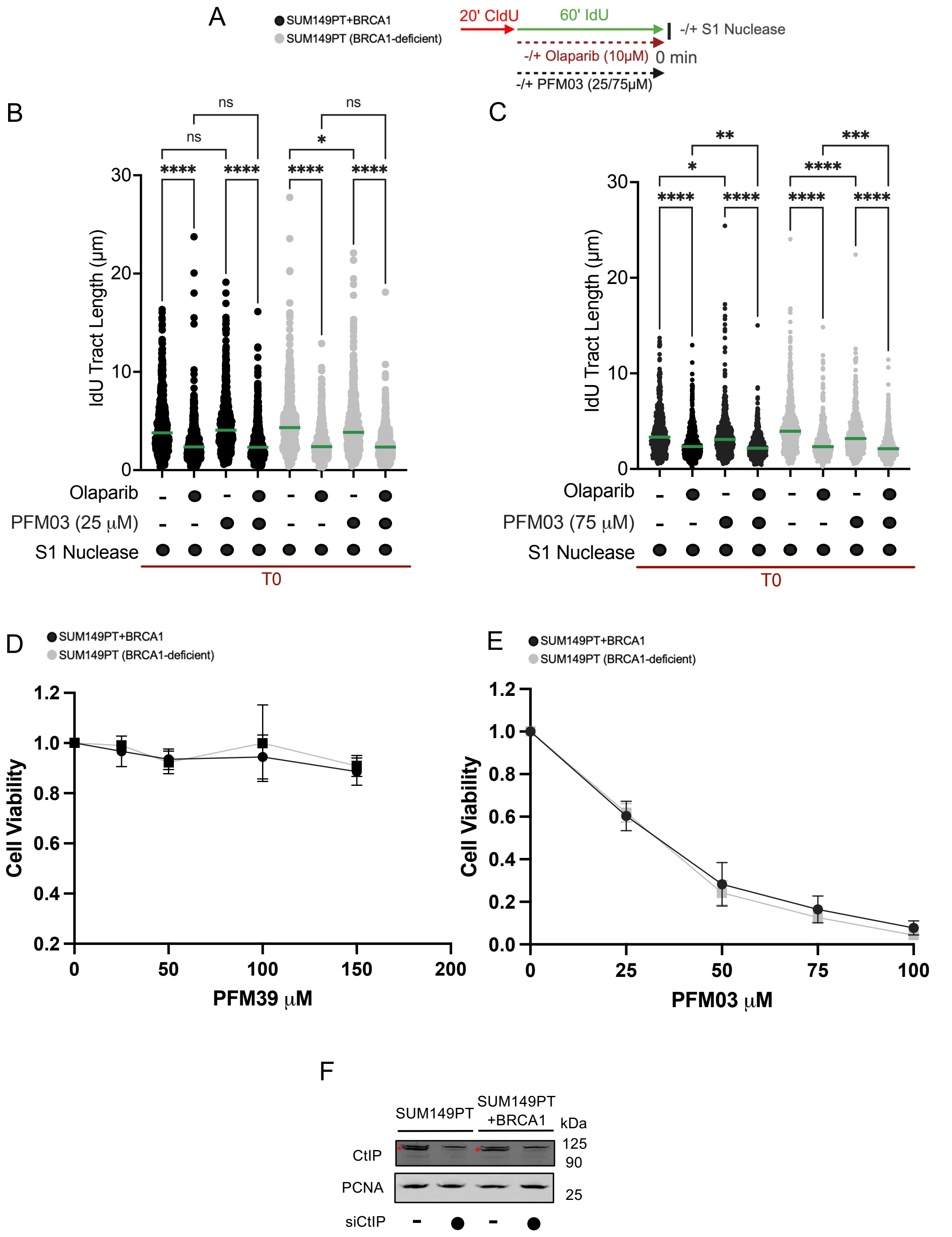

Supplemental Figure S5: Inhibition of MRE11 5'-3' endonuclease activity does not affect ssDNA gap repair in PARPi treated cells (related to Figure 5). (A) Schematic of the DNA fiber spreading assay with the S1 nuclease in the presence and absence of PFM03. (B) Dot plot and median of IdU tract lengths in SUM149PT and SUM149PT+BRCA1 cells treated with the S1 nuclease ± 10 μM Olaparib for 1 h and ± 25 μM PFM03. The S1 nuclease was added immediately after (Time 0) Olaparib removal (*n*=2). At least 180 tracts were scored for each sample. Statistics:

Kruskal-Wallis followed by Dunn's multiple comparisons test. *ns*, non-significant, *p < 0.0332, ****p < 0.0001. (C) Dot plot and median of IdU tract lengths in SUM149PT and SUM149PT+BRCA1 cells treated with the S1 nuclease ± 10 μM Olaparib for 1 h and ± 75 μM PFM03. The S1 nuclease was added immediately after (Time 0) Olaparib removal (*n*=3). At least 150 tracts were scored for each sample. Statistics: Kruskal-Wallis followed by Dunn's multiple comparisons test. *p < 0.0332, **p < 0.0332, ***p < 0.0002, ****p < 0.0001. (D) Cell viability relative to untreated controls in SUM149PT (light grey) and SUM149PT+BRCA1 (black) cells after treatment with increasing concentrations of PFM39 (0, 25, 50, 100 and 150 μM) for 1 h. Mean ±SEM shown. (n=3). Statistics: 2-way ANOVA followed by Bonferonni's multiple comparison test. All comparisons between each cell line at the different PF39 concentrations were *ns*, non-significant. (E) Cell viability relative to untreated controls in SUM149PT (light grey) and SUM149PT+BRCA1 (black) cells after treatment with increasing amounts of PFM03 (0, 25, 50, 100 and 150 μM) for 1 h. Mean ±SEM shown. (n=3). Statistics: 2-way ANOVA followed by Bonferonni's multiple comparison test. All comparisons between each cell line at the different PFM03 concentrations were *ns*, non-significant. (F) Western blot of SUM149PT and SUM149PT+BRCA1 cells depleted for CtIP. Asterisk denotes a specific CtIP band.

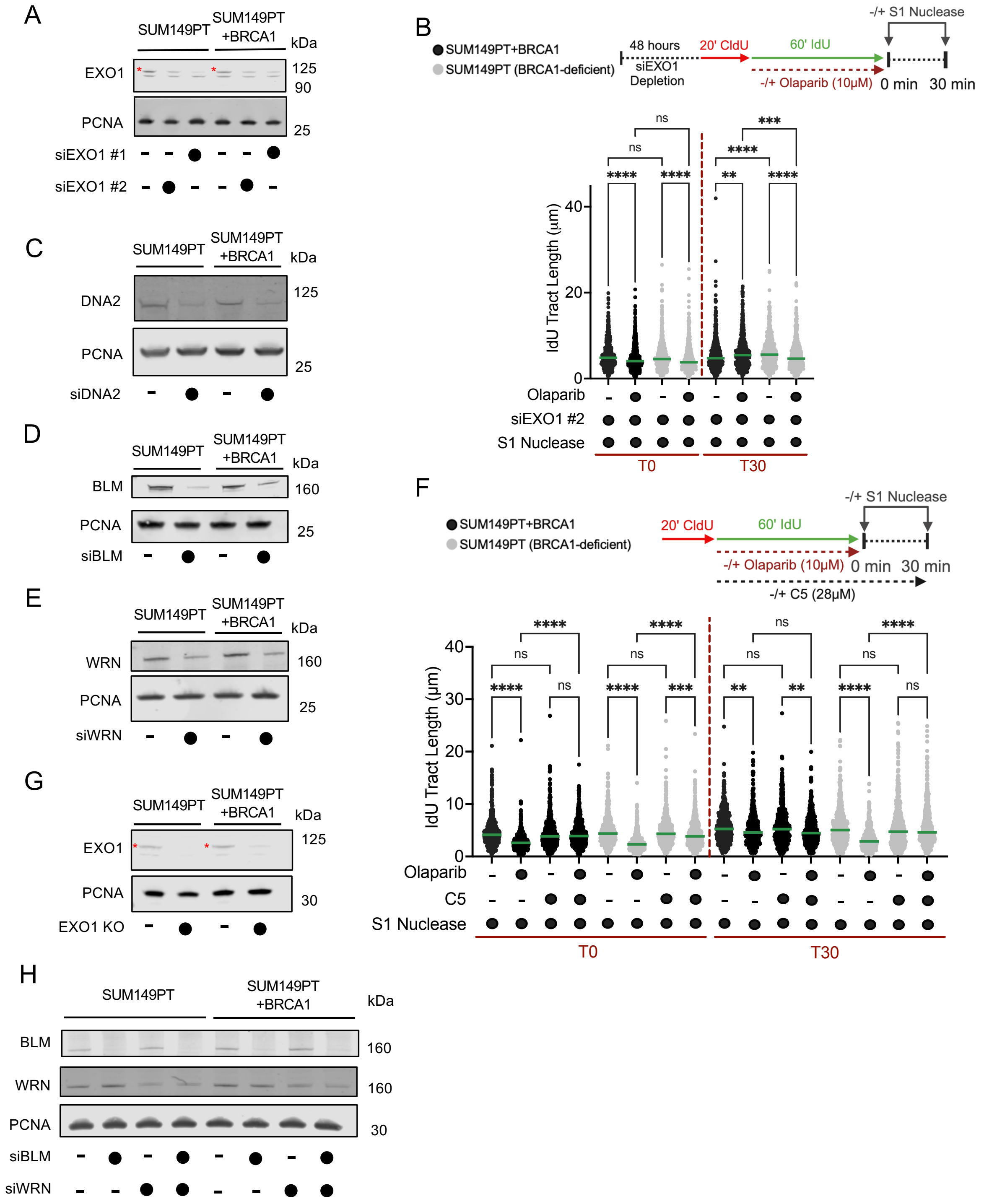

Supplemental Figure S6: EXO1, DNA2, BLM and WRN regulate ssDNA gap repair in PARPi treated cells (related to Figure 6). (A) Western blot of SUM149PT and SUM149PT+BRCA1 cells depleted for EXO1. Asterisk denotes specific EXO1 band. (B) Top, schematic representation of S1 nuclease DNA fiber spreading assay. Bottom, dot plot and median of IdU tract lengths in SUM149PT and SUM149PT+BRCA1 cells treated with the S1 nuclease ± 10 μM Olaparib for 1 h and ± siEXO1 #2. The S1 nuclease was added immediately after (Time 0) and 30 min (Time 30)

after Olaparib removal (*n*=3). At least 180 tracts were scored for each sample. Statistics: Kruskal-Wallis followed by Dunn's multiple comparisons test. *ns*, non-significant, $^{**}p < 0.0021$, $^{***}p < 0.0002$, $^{****}p < 0.0001$. (C) Western blot of SUM149PT and SUM149PT+BRCA1 cells depleted for DNA2. (D) Western blot of SUM149PT and SUM149PT+BRCA1 cells depleted for BLM. (E) Western blot of SUM149PT and SUM149PT+BRCA1 cells depleted for WRN. (F) Top, schematic representation of S1 nuclease DNA fiber spreading assay with C5 inhibitor. Bottom, dot plot and median of IdU tract lengths in SUM149PT and SUM149PT+BRCA1 cells treated with the S1 nuclease ± 10 μM Olaparib for 1 h and ± 28 μM of the DNA2 inhibitor C5. The S1 nuclease was added immediately after (Time 0) and 30 min (Time 30) after Olaparib removal (*n*=3). At least 160 tracts were scored for each sample. Statistics: Kruskal-Wallis followed by Dunn's multiple comparisons test. *ns*, non-significant, $^{**}p < 0.0021$, $^{***}p < 0.0002$, $^{****}p < 0.0001$. (G) Western blot of SUM149PT and SUM149PT+BRCA1 cells with either sgAAVS1 control or pooled sgEXO1 KO. Asterisk denotes specific EXO1 band. (H) Western blot of SUM149PT and SUM149PT+BRCA1 cells depleted for BLM and WRN alone or together.

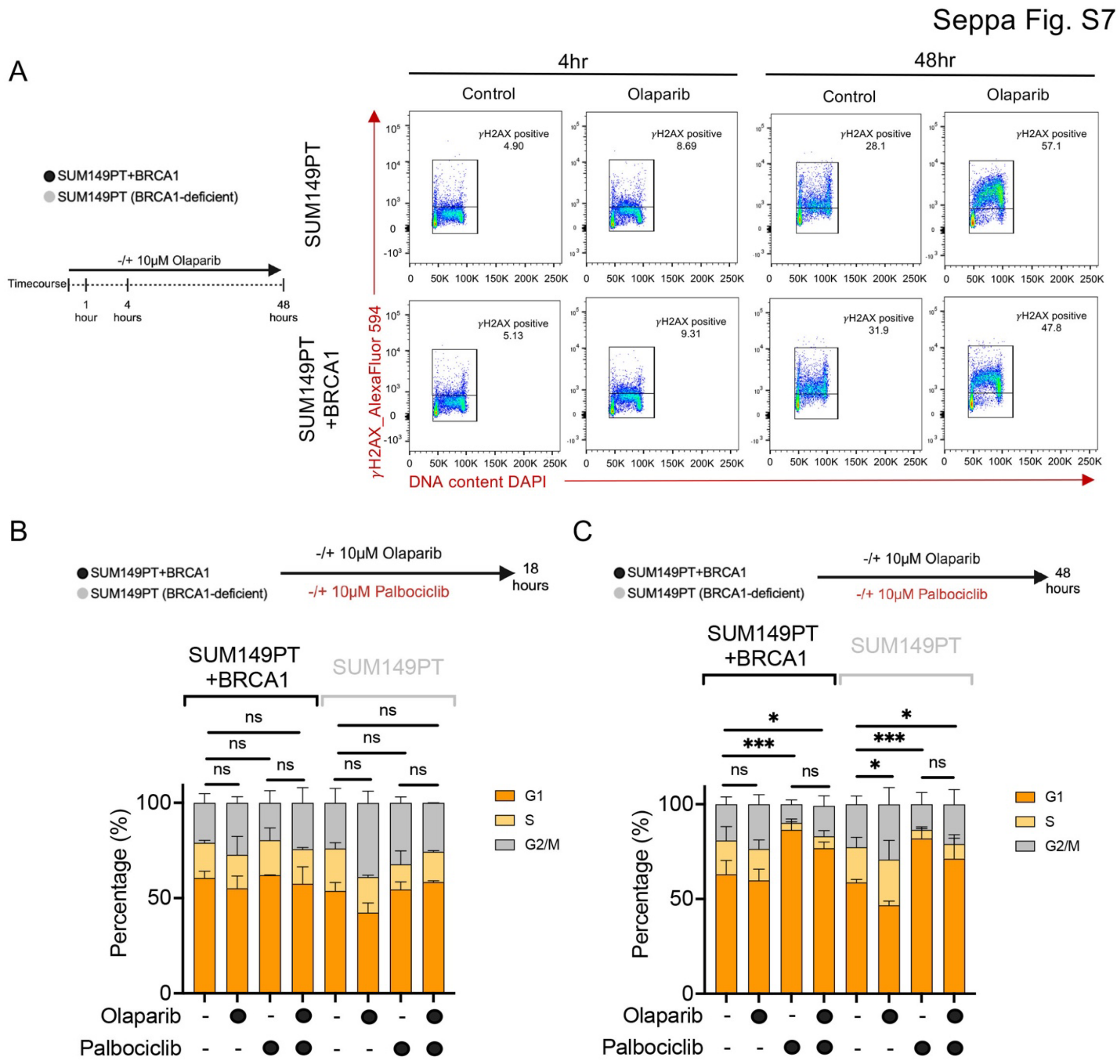

Supplemental Figure S7: DNA breaks only form after prolonged PARPi treatment (related to Figure 7). (A) Left, schematic of γ-H2AX flow cytometry experiment in SUM149PT+BRCA1 and SUM149PT cells. Right, flow cytometry panel for γ-H2AX intensity in SUM149PT+BRCA1 and SUM149PT cells treated with 10 μM Olaparib or DMSO control for 4 or 48 hrs. Both 4- and 48-h panels were normalized to the 1 h treatment conditions. (B) Cell cycle analysis of SUM149PT+BRCA1 and SUM149PT cells treated with 10 μM Olaparib and 10 μM Palbociclib for 18 hrs. Statistical analysis compares cells in the G1 phase. Statistics: One-way ANOVA with Fishers LSD test. *ns*, non-significant. (C) Cell cycle analysis of SUM149PT+BRCA1 and SUM149PT cells treated with 10 μM Olaparib and 10 μM Palbociclib for 48 hrs. Statistical analysis compares cells in the G1 phase. Statistics: One-way ANOVA with Fishers LSD test. *ns*, non-significant, $*p < 0.0332$, $***p < 0.0002$.

Table S1. siRNA sequences. Sequences of the siRNA used for the depletion of BLM, BRCA1, CtIP, DNA2, EXO1, PRIMPOL, and WRN.

Target	Sequence (5' to 3')	Source	Catalog Number
BLM	SMARTpool	Dharmacon	L-007287-02
BRCA1	SMARTpool	Dharmacon	L-003461-00
CtIP	GCUAAAACAGGAACGAAUCUU	Dharmacon	custom made
DNA2	AUAGCCAGUAGUAUUCGAUtt	Ambion	4390827
EXO1 (1)	GGCUAGGAAUGUGCAGACAtt	Ambion	4392420, s17503
EXO1 (2)	CUUUUGAACAGAUCGAUGAtt	Ambion	4392420, s17502
PRIMPOL	GAGGAAACCGUUGUCCUCAGUGUAU	Dharmacon	custom made
WRN	GAUCCAUUGUGUAUAGUUA	Dharmacon	J-010378-05

Table S2. Oligonucleotides use for the biochemical studies. The bold T represents the site of the biotin modification. The asterisk (*) indicates the site of the PTO bonds.

Oligonucleotide Name	Sequence (5' to 3')
X12-4SC	GCGATAGTCTCTAGACAGCATGTCCTAGCAA
X12-4SC-3'-8xPTO	GCGATAGTCTCTAGACAGCATGT*C*C*T*A*G*C*A*A
67bp_compl._X12-3_LONG	GTAATCGTCTATGACGTCTCGAATCATATTTGTAAGAATCAGCTCTG CTCGTGGGATAGTAGGTGTT
66bp_compl._X12-3_LONG	TAATCGTCTATGACGTCTCGAATCATATTTGTAAGAATCAGCTCTGC TCGTGGGATAGTAGGTGTT
63bp_compl._X12-3_LONG	TCGTCTATGACGTCTCGAATCATATTTGTAAGAATCAGCTCTGCTCG TGGGATAGTAGGTGTT
30bp_compl._X12-3_LONG	ATCAGCTCTGCTCGTGGGATAGTAGGTGTT
X12-3_LONG_99bp	AACACCTACTATCCCACGAGCAGAGCTGATTCTTACAAATATGATTC GAGACGTCATAGACGATTACATTGCTAGGACATGCTGTCTAGAGAC TATCGC
5’-10nt	GGTACTGAGGTGGATCTGATCA
3’-10nt	GGTTAGGTGACACTATAGAATA
pSAM-10nt-GAP_1	TCAGTAAGCTTGAATGCTAGTCC
pSAM-10nt-GAP_2	TGAGGACTAGCATTCAAGCTTAC
crRNA-upstream	CTCTTCGCTATTACGCCAGC
crRNA-downstream	TTCCACACAACATACGAGCC
tracrRNA	rGrGrArCrArGrCrArUrArGrCrArArGrUrUrArArArArUrArArGrGrCrUrArGr UrCrCrGrUrUrArUrCrArArCrUrUrGrArArArArArGrUrGrGrCrArCrCrGrAr GrUrCrGrGrUrGrCrUrUrUrU

Supplemental Materials and Methods

Gene silencing with RNAi

Transient gene depletions were done using the Lipofectamine RNAiMax transfection reagent (13778-150, Thermo Fisher Scientific), according to the manufacturer's instruction for 48 hours prior to experiments. The following siRNAs had a final concentration of 40 nM to deplete the gene of interest: BLM (L-007287, Dharmacon), EXO1 (1) (4392420, s17503, Ambion) and EXO1 (2) (4392420, s17502, Ambion). siRNAs for BRCA1 (L-003461, Dharmacon) and WRN (J-010378–05, Dharmacon) had a final concentration of 20 nM. The siRNA transiently depleting DNA2 (4390827, Ambion) had a final concentration of 10 nM and the siRNAs depleting CtIP (custom made, Dharmacon) and PRIMPOL (custom made, Dharmacon) had a final concentration of 50 nM. Silencer select negative control #1 siRNA (4390843, Ambion) was used as control siRNA at the same concentration of the highest siRNA concentration used in each experiment. The sequencing of all the siRNA used in this study is listed in Table S1.

Generation of EXO1 knockout cells

Lentiviral CRISPR guide constructs targeting control AAVS1 (5' GGGGCCACTAGGGACAGGAT3') and EXO1 (5' TCAGGGGGTAGATTGCCTCG 3') were transfected into HEK293 cells using Mirus Transit293 reagent, along with lentiviral packaging vectors pMD2.G (#12259, Addgene) and psPAX2 (#12260, Addgene). Virus was collected 48 and 72 hours post-transfection, pooled, passed through a 0.45 μm filter and stored at −80°C or used immediately. One day prior to infection, cells were plated into 6-well tissue culture plates. Cells were infected with virus in media containing 4 μg/ml polybrene via spin infection Thermo Fisher Sorvall ST16R Centrifuge with an rotor and Microplate Carrier attachment at 650 x g for 30 min. SUM149PT cells were infected with guides cloned into lentiCRISPR v2-blast (#83480, Addgene). Since SUM149PT+BRCA1 cells already expressed a doxycycline-inducible CAS9 construct

under puromycin selection and a sgRNA plasmid targeting BRCA1 used to re-establish BRCA1-proficiency under blast selection, these cells were infected with guides cloned into LRG2.1 (#108098, Addgene) (Wang et al. 2016). The morning following infection, SUM149PT cells were selected in blasticidin for 3 days before media change and propagation of the cell line. For the SUM149PT+BRCA1 cells, the media was changed and 4ug/mL of doxycycline (D5207-10G, Sigma) was added for one week before sorting out the GFP positive population. Subsequent experiments were performed at least three weeks post infection on the pool of cells.

RT-qPCR

Total RNA from experiments was extracted using the PureLink RNA mini Kit (12183018A, Thermo Fisher Scientific). cDNA was made from the RNA by M-MLV Reverse Transcriptase (28025013, Thermo Fisher Scientific) and PCR was conducted using the iQTM SYBR Green supermix (1708880, Biorad) along with the CFX96 Real Time PCR Detection System (Biorad) as indicated by the manufacturers' instructions.

The following primers were used to confirm siRNA depletion:

BRCA1-F 5'AGAAACCACCAAGGTCCAAAG3'

BRCA1-R 5'GGGCCCATAGCAACAGATTT3'

GAPDH-F 5'GAGCCACATCGCTCAGAC3'

GAPDH-R 5'GACCAGGCGCCCAATAC3'

Western blot

Transient gene depletions were confirmed by Western analysis after extracting protein from cell lysates. Lysis buffer containing 9M urea, 75 mM Tris [pH 7.5], and 0.5% of TritonX was used to extract proteins. Protein lysates were sonicated, and total protein concentrations were measured using the Bradford dye (5000205, BIO-RAD) with 10, 5 and 2.5 mg/mL of BSA for a standard

curve. Protein concentrations from 15 to 25 mg were loaded onto either NuPAGE Novex 4-12% Bis-Tris Gel (NP0322BOX, Thermo Fisher Scientific) or 7% Tris-acetate gel (EA0358BOX, Thermo Fisher Scientific). Gels were run with either 1X NuPAGE MES SDS Running Buffer (NP0002, Thermo Fisher Scientific) for the NuPAGE Bis-Tris gels or 1X Tris-Acetate SDS Running Buffer (LA0041, Thermo Fisher Scientific) for 45 minutes at 200V. A 0.45 μM pore nitrocellulose membranes along with transfer buffer containing 1X Tris/Glycine Buffer (1610734, Biorad) with 20% Methanol (10600002, GE Healthcare Life Sciences) were used to transfer proteins from the gels at a constant 400mA for 45 minutes. After transfer the nitrocellulose membranes were blocked in 10% milk (170-6404, Biorad) in TBS-0.1% Tween-20 for 1 hour at room temperature. Incubation with the following primary antibodies was done overnight in BSA in TBS-0.1% Tween-20: Rabbit anti-BLM antibody (1/1,000; A300-110A, Bethyl Laboratories), Mouse anti-BLM (B4) (1/1,000; sc-365753, Santa Cruz Biotechnology), Rabbit anti-CtIP (1/1,000; A300-488A, Bethyl Laboratories) Rabbit anti-DNA2 (1/1,000, ab96488, Abcam), Rabbit anti-Exonuclease1 (1/500, A302-639A, Bethyl Laboratories) Rabbit anti-PRIMPOL (1/1,000; custom made), Mouse anti-PCNA (1/1,000, PC10, sc-56, Santa Cruz) and Rabbit anti-WRN antibody (NB100-471, Novus Biologicals). IRDye Infrared secondary antibodies from LI-COR were used to detect protein expression by the Odyssey CLx (1/20,000, LI-COR) and images were then prepared with Image Studio Lite (LI-COR) (Quinet et al. 2020; Tirman et al. 2021).

Cell Viability Assay

Cell viability assays were performed on SUM149PT and SUM149PT+BRCA1 cells by plating 6,000 cells per well in triplicate in 96-well white-sided clear bottomed plates (655098, Greiner) two days prior to indicated drug treatment with the PFM39 and PFM03 inhibitors for one hour. Cell viability was assessed by Cell Titer Glo (Promega). Plates were scanned using the Infinite 200Pro Reader (Tecan) with Tecan i-control software. Data was normalized to an untreated control for each cell line.

Flow Cytometry

Flow cytometry analysis of γ-H2AX was conducted in SUM149PT and SUM149PT+BRCA1 cells. Cells were treated with either 10μM of Olaparib for 1,4 and 48 hours or with the same amount of DMSO contained in the Olaparib solution for the "untreated" condition. At each time point, cells were trypsinized and washed once with PBS. Pelleted cells were then resuspended in cold 70% ethanol before being vortexed and stored at 20°C for analysis. The day of flow cytometry analysis cells were stained by washing out 70% ethanol solution using 1X cold TBS [pH 7.4] and then rehydrated in cold TST for 10 min at 4°C. The cells in cold tween with salt and Tris (TST) were transferred into a 96 round-bottom plate before being spun down in the plate. The pellet was resuspended and incubated for two hours at 37°C in primary Anti-phospho-Histone H2A.X (Ser139) (1/200; Clone JBW301, 05-636, Millipore Sigma) diluted in TST. After incubation cells were washed two times in TBS before being resuspended and incubated for one hour in the dark at room temperature (RT) in secondary Goat anti-Mouse IgG (H+L) Cross-Absorbed AlexaFluor594 antibody (1/400; A-11005, Thermo Fisher Scientific) diluted in TST. Post incubation cells were washed once with TBS before being incubated at 4°C in the dark for three hours in a staining solution that contains 1 μg/mL DAPI and 100 μg/mL RNaseA diluted in TBS. Stained cells were then flowed using BD FACSymphony™ A3 Cell Analyzer (5 lasers).

Cell Cycle Analysis by Flow Cytometry

For cell cycle analysis, SUM149PT and SUM149PT+BRCA1 cells were treated with 10 μM of Olaparib (PARPi, AZD2281, Selleckchem) concomitant with 10 μM of Palbociclib (PD-0332991) (S1116, Selleckchem) for 48 and 96 hours or with the same amount of DMSO Olaparib plus Palbociclib solutions for the "untreated" condition. After treatment, cells were incubated with 10 μM of 5-ethynyl-2'-deoxyuridine (EdU, E10187, Thermo Fisher Scientific) for 15min, washed twice with PBS, fixed in 70% ethanol, and immediately stored at -20°C. Cells were stained with azide

Alexa Fluor 594 (Click-iT EdU AlexaFluor594 Imaging Kit, C10339) according to the manufacturer's instructions. Cells were stained with analysis buffer (DAPI 1/5,000). Stained cells were then analyzed by cytometry on BD LSRFortessa™ X-20 Cell Analyzer (BD Biosciences).

Expression and purification of recombinant proteins

Human MRE11, the MRN (MRE11-RAD50-NBS1) complex, phosphorylated CtIP (pCtIP), the *S. cerevisiae* MRX (Mre11-Rad50-Xrs2) complex, phosphorylated Sae2 (pSae2), human EXO1, human DNA2, human WRN, human BLM and human RPA were expressed in *Sf*9 insect cells in SFX Insect serum-free medium (Hyclone) using the Bac-to-Bac expression system (Invitrogen), according to manufacturer's recommendations. MRE11 was expressed using the pFB-MBP-MRE11-his vector and was purified by affinity chromatography exploiting the N-terminal maltose-binding protein (MBP)-tag and the C-terminal polyhistidine (his)-tag (Anand et al. 2019). The MBP-tag was removed before the NiNTA affinity purification step using PreScission Protease. The MRN complex was expressed with pTP17 (Tanya Paull, University of Texas at Austin), pFB-RAD50-FLAG and pTP36 (Tanya Paull, University of Texas at Austin), coding for his-tagged MRE11, FLAG-tagged RAD50 and untagged NBS1, respectively, and was prepared using the his-tag at the C-terminus of MRE11 and the FLAG-tag at the C-terminus of RAD50 (Anand et al. 2016). The MRX complex was prepared using a his-tag at the C-terminus of Mre11 and the FLAG-tag at the C-terminus of Xrs2 from cells infected with baculoviruses prepared with pTP391 (Tanya Paull, University of Texas at Austin), pFB-Rad50 and pTP694 (Tanya Paull, University of Texas at Austin), expressing his-tagged Mre11, codon-optimized untagged Rad50 and Xrs2-FLAG, respectively (Cannavo et al. 2013). pCtIP, pSae2, WRN and BLM were expressed using pFB-MBP-CtIP-his, pFB-MBP-Sae2-his, pFB1-MBP_WRN-wt_his, pFB1-MBP_BLM-wt_his, respectively (Cejka and Kowalczykowski 2010; Anand et al. 2016; Pinto et al. 2016; Cannavo et al. 2018). Purification of pCtIP, pSae2, WRN and BLM was performed by affinity chromatography

exploiting the N-terminal MBP-tag and the C-terminal his-tag. The MBP-tag was removed after the amylose purification step using PreScission Protease. For expression of phosphorylated CtIP (pCtIP) and phosphorylated Sae2 (pSae2), *Sf*9 cells were treated with 50 nM or 100 nM Okadaic acid (APExBIO), respectively, for 3 h before cell harvesting, to preserve proteins in their phosphorylated state. 1 µM camptothecin (Sigma) was added for the production of pCtIP to further activate protein phosphorylation cascade 1 h before cell harvesting. Human EXO1 was expressed with pFB-EXO1-FLAG and purified using FLAG affinity chromatography and HiTrap SP HP (Cytiva) ion exchange chromatography (Cannavo et al. 2013; Cannavo et al. 2020). DNA2 was purified by affinity chromatography taking advantage of the N-terminal his-tag and the C-terminal FLAG-tag from cells infected with baculovirus produced using pFB-His-hDNA2-FLAG (Pinto et al. 2016). Human RPA was expressed using the pFB-RPA1, pFB-RPA2 and pFB-6xhis-RPA3 vectors and was purified by NiNTA affinity chromatography, followed by HiTrap Blue column (Cytiva), HiTrap Desalting column (Cytiva) and HiTrap Q column (Cytiva) (Ceppi et al. 2024). Catalytically inactive Cas9 (dCas9) was a kind gift of M. Jinek (University of Zürich) (Jinek et al. 2012; Jinek et al. 2014).

Preparation of oligonucleotide-based DNA substrates

All oligonucleotides were purified by polyacrylamide gel electrophoresis and purchased from Eurogentec. Oligonucleotides were radiolabeled at the 5'-end using T4 polynucleotide kinase (New England Biolabs) and [γ-^{32}P] ATP (Hartmann Analytic) according to manufacturer's instructions. Upon labeling, oligonucleotides were purified on a Micro Bio-Spin P-30 Gel Column (Bio-Rad). The labeled oligonucleotide was then annealed with a 2-fold excess of the respective unlabeled oligonucleotide in annealing buffer (10 mM Tris-HCl pH 8, 50 mM NaCl, 10 mM magnesium chloride), heated to 95°C for 3 min and cooled down to room temperature overnight. To prepare the 1-nt-gapped substrate, X12-4SC, 67bp_compl._X12-3_LONG and X12-3_LONG_99bp oligonucleotides were used. To prepare the 2-nt-gapped substrate, X12-4SC,

66bp_compl._X12-3_LONG and X12-3_LONG_99bp oligonucleotides were used. To prepare the 5-nt-gapped substrate, X12-4SC, 63bp_compl._X12-3_LONG and X12-3_LONG_99bp oligonucleotides were used. To prepare the 40-nt-gapped substrate, X12-4SC, 30bp_compl._X12-3_LONG and X12-3_LONG_99bp oligonucleotides were used. To prepare the 3' recessed substrate, X12-4SC and X12-3_LONG_99bp oligonucleotides were used. To prepared the 1-nt-gapped substrate containing phosphothioate (PTO) bonds X12-4SC-3'-8xPTO, 67bp_compl._X12-3_LONG and X12-3_LONG_99bp oligonucleotides were used. Probes (5'-10 as the 5'-specific probe and 3'-10 as the 3'-specific probe) used for annealing DNA end resection assays were radioactively labeled at the 5' end using T4 polynucleotide kinase (New England Biolabs) and [γ-^{32}P] ATP (Hartmann Analytic). The sequence of all oligonucleotides used in this study is listed in Table S1.

Preparation of plasmid-based DNA substrates

The pSAM-10nt-GAP plasmid used for the *in vitro* assays was generated by ligating annealed and phosphorylated oligonucleotides pSAM-10nt-GAP_1 and pSAM-10nt-GAP_2 (see Table S1 for oligonucleotides sequence) into pG68 digested with BbvCI (New England Biolabs). The 10-nt-gapped version of the substrate was obtained by digestion of pSAM-10nt-GAP with Nb.BsmI (New England Biolabs), followed by digestion with Nb.BbvCI (New England Biolabs). The linear and nicked versions of the substrate were obtained by digestion of pSAM-10nt-GAP with HindIII-HF (New England Biolabs) or Nb.BbvCI (New England Biolabs), respectively. The 68-nt-gapped plasmid was generated by digestion of pG68 with Nb.BbvCI (New England Biolabs). The corresponding linear version of the substrate was obtained by digestion of pG68 with ScaI-HF (New England Biolabs).

Preparation of Cas9-RNPs

The sequences of all oligonucleotides used in this study are listed in Table S1. crRNA and tracrRNA for Cas9 targeting were purchased from Integrated DNA Technologies and annealed at equimolar concentrations (10 μM final) in IDTE buffer pH 8.0 (Integrated DNA Technologies) according to manufacturer's instructions. To produce RNPs, 500 nM (final) catalytically inactive Cas9 (dCas9, a kind gift of M. Jinek, University of Zürich) was incubated with a 3-fold excess of the RNA component (annealed crRNA-tracrRNA) in RNP buffer containing 25 mM Tris-acetate pH 7.5, 5 mM magnesium acetate, 1 mM dithiothreitol (DTT), 0.25 mg/ml bovine serum albumin (BSA, New England Biolabs) and 150 mM KCl for 10 min at 25°C. After incubation, the RNPs were subaliquoted, snap-frozen in liquid nitrogen and stored at -80 °C for later use.

Supplemental References

Anand R, Jasrotia A, Bundschuh D, Howard SM, Ranjha L, Stucki M, Cejka P. 2019. NBS1 promotes the endonuclease activity of the MRE11-RAD50 complex by sensing CtIP phosphorylation. *EMBO J* **38**.

Anand R, Ranjha L, Cannavo E, Cejka P. 2016. Phosphorylated CtIP Functions as a Co-factor of the MRE11-RAD50-NBS1 Endonuclease in DNA End Resection. *Mol Cell* **64**: 940-950.

Cannavo E, Cejka P, Kowalczykowski SC. 2013. Relationship of DNA degradation by Saccharomyces cerevisiae exonuclease 1 and its stimulation by RPA and Mre11-Rad50-Xrs2 to DNA end resection. *Proc Natl Acad Sci U S A* **110**: E1661-1668.

Cannavo E, Johnson D, Andres SN, Kissling VM, Reinert JK, Garcia V, Erie DA, Hess D, Thoma NH, Enchev RI et al. 2018. Regulatory control of DNA end resection by Sae2 phosphorylation. *Nat Commun* **9**: 4016.

Cannavo E, Sanchez A, Anand R, Ranjha L, Hugener J, Adam C, Acharya A, Weyland N, Aran-Guiu X, Charbonnier JB et al. 2020. Regulation of the MLH1-MLH3 endonuclease in meiosis. *Nature* **586**: 618-622.

Cejka P, Kowalczykowski SC. 2010. The full-length Saccharomyces cerevisiae Sgs1 protein is a vigorous DNA helicase that preferentially unwinds holliday junctions. *J Biol Chem* **285**: 8290-8301.

Ceppi I, Dello Stritto MR, Mutze M, Braunshier S, Mengoli V, Reginato G, Vo HMP, Jimeno S, Acharya A, Roy M et al. 2024. Mechanism of BRCA1-BARD1 function in DNA end resection and DNA protection. *Nature*.

Jinek M, Chylinski K, Fonfara I, Hauer M, Doudna JA, Charpentier E. 2012. A programmable dual-RNA-guided DNA endonuclease in adaptive bacterial immunity. *Science* **337**: 816-821.

Jinek M, Jiang F, Taylor DW, Sternberg SH, Kaya E, Ma E, Anders C, Hauer M, Zhou K, Lin S et al. 2014. Structures of Cas9 endonucleases reveal RNA-mediated conformational activation. *Science* **343**: 1247997.

Pinto C, Kasaciunaite K, Seidel R, Cejka P. 2016. Human DNA2 possesses a cryptic DNA unwinding activity that functionally integrates with BLM or WRN helicases. *Elife* **5**.

Quinet A, Tirman S, Jackson J, Svikovic S, Lemacon D, Carvajal-Maldonado D, Gonzalez-Acosta D, Vessoni AT, Cybulla E, Wood M et al. 2020. PRIMPOL-Mediated Adaptive Response Suppresses Replication Fork Reversal in BRCA-Deficient Cells. *Mol Cell* **77**: 461-474 e469.

Tirman S, Quinet A, Wood M, Meroni A, Cybulla E, Jackson J, Pegoraro S, Simoneau A, Zou L, Vindigni A. 2021. Temporally distinct post-replicative repair mechanisms fill PRIMPOL-dependent ssDNA gaps in human cells. *Mol Cell* **81**: 4026-4040 e4028.

Wang Y, Bernhardy AJ, Cruz C, Krais JJ, Nacson J, Nicolas E, Peri S, van der Gulden H, van der Heijden I, O'Brien SW et al. 2016. The BRCA1-Delta11q Alternative Splice Isoform Bypasses Germline Mutations and Promotes Therapeutic Resistance to PARP Inhibition and Cisplatin. *Cancer Res* **76**: 2778-2790.
